# Identification of potential biomarkers related to the Bitong mixture in osteoarthritis based on bioinformatics and network pharmacology, and exploration of the mechanism involved

**DOI:** 10.3389/fimmu.2026.1739355

**Published:** 2026-02-13

**Authors:** Ziwei Shen, Suming Li, Teng Li, Lining Wang

**Affiliations:** 1 Department of Orthopedics and Traumatology, Nanjing Hospital of Traditional Chinese Medicine, Nanjing, Jiangsu, China; 2 Department of Orthopedics and Traumatology, School of Integrated Traditional Chinese and Western Medicine, Nanjing University of Chinese Medicine, Nanjing, Jiangsu, China

**Keywords:** osteoarthritis, Bitong mixture, biomarkers, single-cell analysis, bioinformatics, network pharmacology, molecular docking, RT-qPCR

## Abstract

**Background:**

Bitong Mixture (BM) has shown efficacy in alleviating pain in knee osteoarthritis (OA) in clinical practice; however, the molecular mechanisms underlying its therapeutic effects remain to be fully elucidated. This study aimed to identified BM-related OA biomarkers and explore their functional implications.

**Methods:**

An integrative strategy combining bioinformatics prediction and experimental validation was used. Biomarkers were screened from public OA transcriptomic data using differential expression analysis, network pharmacology, and machine learning. Their functions were explored via enrichment and immune infiltration analyses. Molecular docking predicted interactions between herbal compounds and targets. Single-cell analysis characterized biomarker expression in chondrocyte subsets. A rat OA model and reverse transcription quantitative polymerase chain reaction (RT-qPCR) were employed for *in vivo* validation.

**Results:**

Bioinformatic prediction identified three potential biomarkers: MMP9, MMP2, and SPP1. They demonstrated certain diagnostic performance for OA and were implicated in pathways related to extracellular matrix organization and immune regulation. Immune analysis revealed significant correlations, notably between MMP2 and activated dendritic cells (cor = 0.66) and between SPP1 and CD4+ central memory T cells (cor = -0.75). Molecular docking suggested strong binding affinity between luteolin (a BM component) and MMP9. Single-cell analysis indicated high expression of these potential biomarkers in hypertrophic chondrocytes, inflammatory chondrocytes, and fibrochondrocytes. *In vivo* validation confirmed that BM alleviated OA symptoms and histopathological damage in rats. RT-qPCR results showed that BM treatment alleviated the OA-induced upregulation of MMP9, MMP2, and SPP1 expression.

**Conclusion:**

MMP9, MMP2, and SPP1 are potential therapeutic biomarkers for BM in OA. The efficacy of BM may be attributed to its regulation of extracellular matrix remodeling and immune responses, which provides a possible mechanistic explanation for its clinical use.

## 1 Introduction

Osteoarthritis (OA) is a chronic degenerative joint disorder characterized by progressive cartilage destruction, synovial inflammation, and subchondral bone sclerosis (1). This multifaceted disease represents one of the most prevalent musculoskeletal conditions worldwide, significantly impacting quality of life and imposing substantial socioeconomic burdens on healthcare systems globally (2). The pathogenesis of OA involves complex multifactorial interactions, including aging, mechanical overload, metabolic dysfunction (e.g., obesity), genetic susceptibility, and inflammatory cascades that perpetuate tissue damage (3–5). These interconnected pathways contribute to the heterogeneous nature of OA, making it challenging to develop universally effective therapeutic interventions (6). Epidemiologically, OA affects over 300 million people globally, with a disproportionate impact on postmenopausal women and the elderly population, demonstrating rising prevalence due to aging demographics and increasing obesity rates (7). The disease burden is projected to escalate further as life expectancy increases and sedentary lifestyles become more prevalent. Current therapeutic approaches, including nonsteroidal anti-inflammatory drugs (NSAIDs), intra-articular corticosteroids, hyaluronic acid injections, and joint replacement surgery, primarily focus on symptom management rather than addressing underlying disease mechanisms, thus failing to halt or reverse disease progression (8). Notably, chronic NSAID administration increases risks of peptic ulcers, cardiovascular complications, and renal impairment, while surgical options are limited by prosthesis longevity, postoperative complications, and patient-specific factors (9, 10). These therapeutic limitations underscore the urgent need to identify novel prognostic biomarkers and innovative therapeutic targets to better elucidate OA pathogenesis and provide more effective approaches for clinical diagnosis, prognosis assessment, and targeted treatment strategies.

Traditional Chinese medicine (TCM), a comprehensive holistic therapeutic system encompassing herbal compounds, acupuncture, manual therapies, and dietary interventions, has garnered increasing scientific attention for its multi-target efficacy and remarkably low toxicity profile in managing chronic diseases, including OA (11). This ancient medical paradigm operates on the principle of restoring balance within the body’s energy systems while addressing root causes rather than merely symptomatic relief. TCM formulations, systematically classified into standardized patent medicines and individualized customized decoctions, demonstrate remarkable therapeutic versatility by modulating complex pathophysiological processes, including inflammation, oxidative stress, cartilage metabolism, and immune dysfunction, through sophisticated synergistic interactions of multiple bioactive components (12–14). Recent pharmacological investigations have highlighted the significant therapeutic potential of specific TCM herbs in OA management, with Curcuma longa exhibiting potent anti-inflammatory properties through NF-κB pathway inhibition, and Eucommia ulmoides demonstrating cartilage-protective effects via enhanced collagen synthesis and chondrocyte proliferation (15). These findings validate the traditional empirical knowledge with modern scientific evidence. The Bitong mixture (BM) is a hospital-prepared medicine of Nanjing Hospital of Traditional Chinese Medicine (Approval No.: Su Yao Zhi Zi Z04000864). It has been clinically used for the treatment of osteoarthritis for decades and achieved favorable therapeutic outcomes., It is a classical TCM formula comprising Aconitum carmichaelii, Paeonia lactiflora, and multiple complementary herbs, has demonstrated significant analgesic and anti-arthritic effects in preliminary clinical observations and experimental studies (16–18). Despite these promising therapeutic outcomes, the specific bioactive compounds, precise molecular targets, and underlying mechanisms responsible for BM’s OA improvement remain poorly characterized and inadequately understood. Therefore, systematic investigation of BM’s comprehensive pharmacological basis is imperative to bridge the gap between traditional empirical knowledge and contemporary evidence-based clinical application, facilitating its integration into modern therapeutic protocols.

In this study, We adopted a strategy integrating bioinformatics, network pharmacology, and experimental verification to screen candidate biomarkers related to BM treatment of OA, predict their potential molecular targets and pathways, and conduct preliminary validation through animal models and molecular experiments, thereby providing clues and hypotheses for the subsequent in-depth mechanism study and clinical application of BM.

## 2 Materials and methods

### 2.1 Study design and workflow

To identify potential biomarkers and explore the therapeutic mechanisms of BM in OA, wepinpointed candidate biomarkers through data mining, experimentally validated their relevance and BM’s *in vivo* efficacy, and conducted mechanistic and cellular-scale analysis to further dissect the underlying contexts (**Supplementary Figure 1**).

### 2.2 Establishment of osteoarthritis rat model

In this experiment, a total of 15 8-week-old male Sprague-Dawley (SD) rats weighing 250 g were obtained from SPF(Beijing)BIOTECHNOLOGY Co., Ltd. These rats were first subjected to one week of adaptive feeding. The sample size was determined with reference to similar pharmacological studies of OA (19) and based on a sample size calculation using G*Power software, which indicated that a minimum of 4 animals per group was required to achieve statistical significance (effect size d = 2.0, α = 0.05, β = 0.8). To ensure robust statistical power, five rats per group were ultimately adopted in this study. Additionally, the monosodium iodoacetate (MIA) model was selected for its ability to rapidly and reproducibly induce key OA pathological features including cartilage degradation, synovitis, and pain behavior, which is suitable for short-term intervention studies and has been widely used in OA drug screening and mechanistic research (20). Subsequently, 15 rats were selected and randomly divided into 3 groups: the Control group, the OA group, and the OA+Arthralgia Compound group. Except for the rats in the Control group, the rats in other groups were anesthetized with 2.5-3% isoflurane. An 8% sodium iodoacetate solution was prepared (2 mg of sodium iodoacetate was dissolved in 50 μl of normal saline). With the rat’s knee joint slightly flexed, a 1-ml insulin syringe was inserted slightly medial to the patella and punctured obliquely upward and medially. When a distinct sense of giving way was felt, indicating that the needle tip had pierced into the joint cavity, the corresponding volume of sodium iodoacetate solution was injected into the bilateral knee joint cavities of each rat to establish the model. Intervention began 14 days after model establishment. For the rats in the OA + BM group, they were intragastrically administered with the Bitong mixture (BM) (containing various traditional Chinese medicine components such as Caulis Sargentodoxae 1.35g/kg, Radix Saposhnikoviae 0.9g/kg, Processed Aconitum carmichaeli Debx. 0.9g/kg, Processed Aconitum kusnezoffii Reichb. 0.9g/kg, Radix Angelicae Pubescentis 0.9g/kg, Radix Dipsaci 1.08g/kg, Radix Achyranthis Bidentatae 1.35g/kg, Rhizoma Cibotii 0.9g/kg, Rhizoma Polygoni Cuspidati 1.35g/kg, Radix Glycyrrhizae 0.9g/kg, Stir-fried Rhizoma Atractylodis Macrocephalae 1.08g/kg, Semen Coicis 1.35g/kg, Radix Clematidis 0.9g/kg, Pericarpium Citri Reticulatae 0.9g/kg, Cortex Periplocae 0.9g/kg, Eupolyphaga Seu Steleophaga 0.54g/kg, Radix Cynanchi Paniculati 0.9g/kg, Poria 1.35g/kg, Ramulus Cinnamomi 0.9g/kg) at a dose of 19.35 g/kg, which was derived from the clinical human dose (assuming a body weight of 70 kg) using a human-to-rat equivalent dose conversion factor of 6.3 based on body surface area (21), once a day for 14 consecutive days. BM is a standardized hospital preparation produced by Nanjing Hospital of Traditional Chinese Medicine with the approval number [Su Yao Zhi Zi Z04000864]. Its production strictly complies with the quality standards outlined in the *Pharmacopoeia of the People’s Republic of China* (22) and the *Good Preparation Practice for Pharmaceutical Preparations in Medical Institutions* (https://www.nmpa.gov.cn/yaopin/ypfgwj/ypfgbmgzh/20010313010101514.html), ensuring regulatory oversight and clinical applicability. To guarantee batch-to-batch consistency, the manufacturing process follows established standardized operating procedures. Key steps include soaking multiple Chinese herbal pieces in water, decoction twice, combining filtrates, allowing sedimentation, concentrating, adding flavoring and preservative agents, filtering, filling, and sterilizing, thereby ensuring both chemical stability and pharmacological consistency between batches. The weight and Lequesne MG score were collected to assess the treatment effect of BM in OA. The articular cartilage tissue samples and serum samples of 15 experimental rats were collected for subsequent experiments. All experimental procedures involving animals were approved by the Institutional Animal Care and Use Committee (IACUC).

### 2.3 Histological staining

After the articular cartilage tissue of 15 rats was removed, it was decalcified using an ethylenediaminetetraacetic acid (EDTA) decalcification solution. After the decalcification was completed, the tissue was taken out, washed thoroughly with phosphate-buffered saline (PBS), and then immersed in a 4% paraformaldehyde solution for fixation. Subsequently, the tissue was dehydrated successively using 70% ethanol, 80% ethanol, 95% ethanol, and 100% ethanol. After the dehydration steps were finished, the tissue was soaked in a mixture of xylene and absolute ethanol until it became transparent. Finally, the transparent tissue was placed in paraffin for soaking and infiltration.

In this experiment, hematoxylin and eosin (H&E) staining was performed on tissue samples from three groups. The 5-μm sliced tissue was placed in alcohol for flattening. After it was flattened, the sliced tissue on the slide was put into an oven for baking. Once the paraffin melted, the slide was taken out and immersed in xylene for dewaxing. Subsequently, the slide was hydrated successively with 100% alcohol, 95% alcohol, 80% alcohol, and 70% alcohol, and then rinsed with PBS solution. Next, the slide was stained with hematoxylin and then rinsed with distilled water. It was then put into an alcohol-hydrochloric acid solution for differentiation and placed in tap water to make it turn blue again. After that, the slide was stained with eosin and rinsed in distilled water. Subsequently, it was dehydrated successively with 95% alcohol and 100% alcohol, and then soaked in xylene until it became transparent. Finally, the slide was sealed with neutral balsam.

Similar to the H&E staining, the sliced tissue with a 3-μm sliced tissue was stained with safranin. Then it was stained with Weigert staining solution and differentiated with an acidic differentiating solution. Subsequently, it was immersed in fast green staining solution. After the immersion, the slice was washed with a weak acidic solution, and then it was immersed in safranin staining solution again. After that, it was successively soaked in alcohol for dehydration and in xylene until it reached a transparent state. Finally, it was sealed with optical resin.

The histological scoring of all tissue sections was performed under blinded conditions. Specifically, all tissue sections were assigned anonymous numerical codes prior to evaluation. The researcher responsible for the histological assessment was unaware of the group assignments (Control, OA, or OA+BM) of each sample throughout the entire scoring process. The codes were only revealed and linked to the experimental groups after the analysis was completed.

### 2.4 Data acquisition

In this study, three OA-related human datasets were downloaded from the Gene Expression Omnibus (GEO) database (https://www.ncbi.nlm.nih.gov/gds). Specifically, GSE114007 (GPL11154) included 18 normal (control group) and 20 OA (case group) human knee cartilage tissue samples, and GSE169077 (GPL96) comprised 5 normal and 6 OA knee cartilage tissue samples. Additionally, the single-cell dataset GSE255460 (GPL24676) consisted of 16 OA and 3 human knee cartilage tissue samples. Among them, GSE114007 and GSE169077 were used as the training set and validation set, respectively. The classification criteria were relatively consistent across datasets, and sample classification in all datasets adhered to the original studies’ diagnostic criteria. Batch correction was not performed since GSE114007 and GSE169077 were analyzed separately as independent training and validation sets, and GSE255460 was processed independently as a single-cell dataset without merging into a unified expression matrix. The detailed characteristics of these datasets, including species, tissue source, disease stage, and platform, are provided in **Supplementary Table 1**.

### 2.5 Differential expression and enrichment analysis

In the human OA transcriptomic dataset GSE114007, differential expression analysis was employed to screen for differentially expressed genes (DEGs) between case and control groups by DEseq2 package (v 1.34.0) (19), the results were presented in volcano maps and heat maps by ggplot2 (v 3.3.6) (20) and pheatmap3 (v 1.1.9) (21) packages, respectively. Briefly, raw read counts were preprocessed by removing genes with an average expression below 0.5. A pseudocount of 1 was added to all counts for subsequent log transformation. Differential expression analysis was then conducted using DESeq2 based on a negative binomial generalized linear model. The design matrix was constructed with the sample condition (OA vs. Control) as the variable. Gene-wise dispersion estimates were calculated and a model was fitted using the DESeq() function. The Benjamini-Hochberg procedure was applied to adjust P-values for multiple testing control. DEGs were identified based on thresholds of |log2 fold change (FC)| ≥ 1 and an adjusted p-value (false discovery rate, FDR) < 0.05 (23).

Meanwhile, drug targets corresponding to active compounds in BM (oral bioavailability (OB) ≧ 30% and drug likeness (DL) ≧ 0.18) (24, 25) were predicted in the Traditional Chinese Medicine Systems Pharmacology (TCMSP) database (https://tcmsp-e.com/tcmsp.php). After that, the drug targets obtained above were intersected with the human OA-related DEGs to further identify candidate genes. Subsequently, the biological functions and pathways associated with candidate genes were explored through the clusterProfiler package (v 4.8.2) (22), which included Gene Ontology (GO) and Kyoto Encyclopedia of Genes and Genomes (KEGG) enrichment analyses (adj. p < 0.05) (26).

### 2.6 Construction of protein-protein interaction network

To further explore the interactions among the human OA-derived candidate genes at the protein level, their PPI network was constructed using the STRING database (http://string.embl.de/) (confidence score > 0.4) (27). Subsequently, the top 1 connectivity gene cluster was selected using the MCODE plugin. Next, hub genes in the top 1 gene cluster were further identified using 3 algorithms (Degree, Betweenness, Closeness) in the cytoHubba plugin. The intersection of hub genes identified by these 3 algorithms was employed to mine candidate key genes.

### 2.7 Machine learning analysis

Firstly, the human OA-derived candidate key genes were subjected to least absolute shrinkage and selection operator (LASSO) regression analysis according to the glmnet package (v 4.1-2) (23), where 10-fold cross validation was used to identify feature genes 1. Meanwhile, the Boruta algorithm was also used to screen the feature genes 2. The key feature genes were obtained by taking the intersection of feature genes from these two machine learning algorithms. Subsequently, the expression levels of key feature genes were investigated in the human OA dataset GSE114007 and GSE169077 using the Wilcoxon test (p < 0.05) (28). The genes showing significant differences in case and control groups and consistent expression trends across both datasets were considered potential biomarkers. Following this, receiver operating characteristic (ROC) curves for these potential biomarkers were plotted in both datasets using the pROC package (v 1.18.0) (24) to further evaluate their diagnostic performance for OA (area under curve (AUC) values > 0.7) (29).

### 2.8 Construction of a nomogram

In order to further explore the predictive performance of potential biomarkers for OA patients, a potential biomarker nomogram was constructed, where the occurrence of OA was predicted according to the total points corresponding to each potential biomarker. After that, the accuracy of the nomogram model prediction was further evaluated by calibration curve, decision curve analysis (DCA), and ROC curve.

### 2.9 Functional analysis of potential biomarkers

In the human OA dataset GSE114007, potential biomarkers related to signaling pathways were explored. Specifically, the correlation of potential biomarkers with other genes was calculated separately and sequenced. After that, the human C2: KEGG gene set (Homo sapiens) was downloaded through the msigdbr package (v 7.5.1) (30) as the background set. Afterwards, the sequenced genes were subjected to gene set enrichment analysis (GSEA) by the GSEA function (adj. p < 0.05). In addition, in order to explore the roles played by potential biomarkers *in vivo* as well as the associated interacting proteins, GeneMANIA (http://genemania.org) was employed to construct the gene-gene interaction (GGI) network of potential biomarkers, where the top 20 genes and the top 7 signaling pathways were presented.

### 2.10 Immune-related analyses

To understand whether there was a significant difference in the enrichment of immune cells during the progression of OA compared with normal people, xcell (v 1.1.0) (31) was used to perform immune cell infiltration analysis in the human OA dataset GSE114007. Besides, the infiltration scores of 64 immune cells were obtained, samples that were significantly enriched in immune cells were screened, and all immune cells that were not enriched in the samples were removed. Thereafter, the Wilcoxon test was used to analyze the difference in immune cells enrichment scores between the case and control groups (p < 0.05). Eventually, the relationship between potential biomarkers and 64 immune cells was revealed by the Spearman method.

### 2.11 Regulatory network analysis

In order to deeply explore the expression regulation mode of potential biomarkers, their regulatory networks were constructed. Firstly, miRNAs that may regulate potential biomarker expression were predicted using the miRDB database (http://www.mirbase.org). Among them, the top 10 miRNAs were used for lncRNA prediction in the Starbase database (http://starbase.sysu.edu.cn)(pancancerNum≧10). After that, the top 5 lncRNAs sorted by pancancerNum associated with each miRNA were used for lncRNA-miRNA-mRNA network construction via Cytoscape (v 3.9.1) (32). Also, the transcription factors (TFs) of the potential biomarkers were predicted in the CistromeDB database (Http://cistrome.org/db).

### 2.12 Molecular docking

In this study, the herbal-active compounds-gene - gene network of potential biomarkers was constructed via Cytoscape software (v 3.9.1) (32). Besides, the common targets of the compounds were selected for molecular docking. In detail, mRNA-encoded proteins in PDB format files were downloaded from Uniprot Protein Database (https://www.uniprot.org), while SDF format files of components were obtained from the PubChem database (https://pubchem.ncbi.nlm.nih.gov/). After that, the drug active compounds and gene active compounds results were pretreated with the CB-DOCK2 tool (33). Finally, the molecular docking of the active compounds of the drug with the mRNA-encoded proteins was performed via AutoDock (v 1.1.2) (34), and the results were visualized via PyMol (v2.4.1) (35).

### 2.13 Single-cell analysis

After completing the molecular docking analysis, the GSE255460 dataset (human OA single-cell RNA-seq) was further analyzed using the Seurat package for single-cell RNA sequencing data. First, low-quality cells were filtered out based on quality control criteria: only cells with nFeature_RNA between 200 and 4,000, nCount_RNA below 20,000, and percent.mt under 15% were retained for further analysis. The retained data was then normalized using the “LogNormalize” method. After log normalization, the “FindVariableFeatures” function with the “vst” method was used to identify the top 2,000 highly variable genes to optimize computational efficiency. Next, principal component analysis (PCA) was conducted on individual samples using the “RunPCA” function, and sample integration was achieved using the “CCAIntegration” method within the “IntegrateLayers” function. After PCA dimensionality reduction, unsupervised clustering was performed using “FindNeighbors” and “FindClusters” functions in “Seurat” (v 5.0.1) (36) (resolution = 0.5) (37). Subsequently, cell annotation was performed based on the TSNE clustering results and marker genes provided in the literature (38). The distribution of potential biomarkers across cell clusters was visualized. Finally, the “CellChat” (v 1.6.1) (39) was employed to analyze cell-to-cell communication at the molecular level. The number of receptor-ligand pairs was counted, and chord diagrams were employed to visualize the communication and regulatory networks between cells.

### 2.14 Reverse transcription-quantitative PCR

Because articular cartilage is the primary site of degeneration in osteoarthritis, it was selected for RT-qPCR analysis to directly evaluate gene expression changes at the disease epicenter. Articular cartilage tissues were harvested from the rat models described in Section 2.1. RNAs of 5 control, 5 OA, and 5 treatment (using BM to treat OA) group articular cartilage tissue samples were isolated via TRIzol reagent (Ambion, America). After being detected by electrophoresis and analyzed for purity on NanoPhotometer N50, RNAs were subjected to cDNA synthesis using the SureScript-First-strand-cDNA-synthesis kit (Servicebio, China). Then RT-qPCR was conducted by Universal Blue SYBR Green qPCR Master Mix (Servicebio, China) on a CFX connect RT-qPCR detection system (BIO-RAD, America). Primers of potential biomarkers and internal reference gene (GAPDH) were shown in **Supplementary Table S1**. Besides, the reaction system and program for reverse transcription and RT-qPCR were shown in **Supplementary Tables S2**-**S5**. After RT-qPCR reaction, the 2^-ΔΔCт^ method was employed to calculate relative expression. Eventually, t-test (P < 0.05) (40) was conducted to assess intergroup differences, and Graphpad Prism 5 software (v 8.0) (41) was applied for visualization.

### 2.15 Statistical analysis

Bioinformatic analyses were conducted in the R program (v 4.2.2). Additionally, employing the Wilcoxon rank sum test or t-test to determine group differences, with a significance threshold defined at P < 0.05.

## 3 Results

### 3.1 BM alleviates OA symptoms and joint damage (rat model)

**Figure 1:**
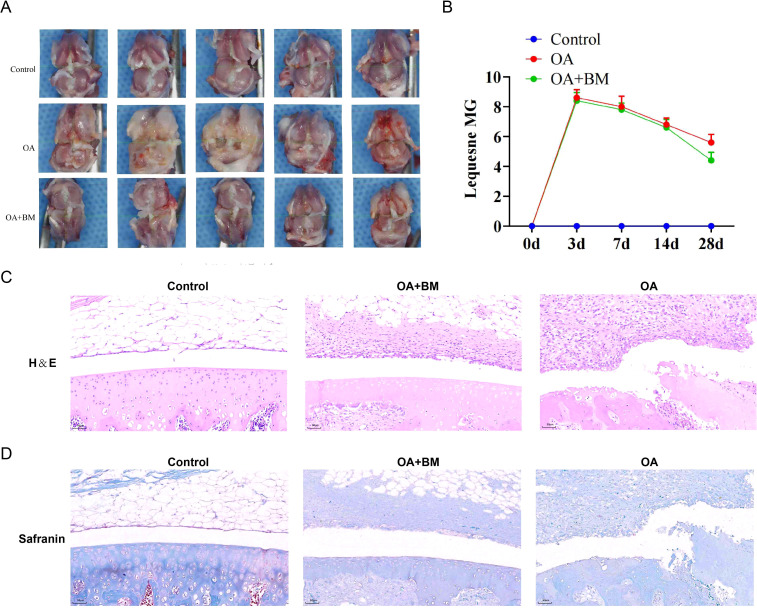
**(A)** Three groups of rat knee joint photos, showing significant hyperplasia and inflammatory phenotype in the OA group knees. **(B)** In the line chart of Lequesne MG score of the three groups over time, the score of the 3–7 days OA group was the highest, and the score of the 28 days OA + Bitong mixture group was significantly decreased. **(C)** H&E staining images of articular cartilage tissues in three groups of rats. Compared with the control group and the OA+BM group, in the OA group, the cartilage tissue proliferates, the number of cell layers increases, and the fibrous tissue thickens. ((1:control, 2:OA+BM, 3:OA). **(D)** Safranin staining images of articular cartilage tissues in three groups of rats, Compared with the control group and the OA+BM group, Cartilage loss was severe in the OA group(1:control, 2:OA+BM, 3:OA).

To initially validate the therapeutic efficacy of BM, we assessed its effects on pain behavior and joint histopathology in an MIA-induced rat OA model. In the animal model, the knee joints of the rat samples in the OA group exhibited obvious hyperplasia and inflammatory phenotypes when compared with those in the control group and the OA + BM group (**Figure 1A**). It is speculated that this was caused by the excessive infiltration of inflammatory cells. In addition, the Lequesne MG scores of the three groups of animal models showed that the Lequesne MG scores reached the highest level from 3 to 7 days after the injection of sodium iodoacetate. This indicated that the damage caused by sodium iodoacetate to the rats was most significant during this 3- to 7-day period. However, the scoring results on the 28th day showed that after the intervention of the OA+BM group, the scores decreased significantly (**Figure 1B**). These results suggested that BM can effectively alleviate the damage caused by OA. Furthermore, the results of H&E staining and safranin staining showed that, compared with the control group and the OA+BM group, the articular cartilage tissue of the samples in the OA group exhibited hyperplasia, an increase in the number of cell layers, thickening of the fibrous tissue, indicating a large number of infiltrating inflammatory cells, and severe cartilage loss (**Figures 1C, D**). These results further indicated at the histopathological level that BM could alleviate the symptoms of OA and have a protective effect on joint tissue.

### 3.2 Identification and functional enrichment of 64 candidate genes (human: GSE114007)

**Figure 2:**
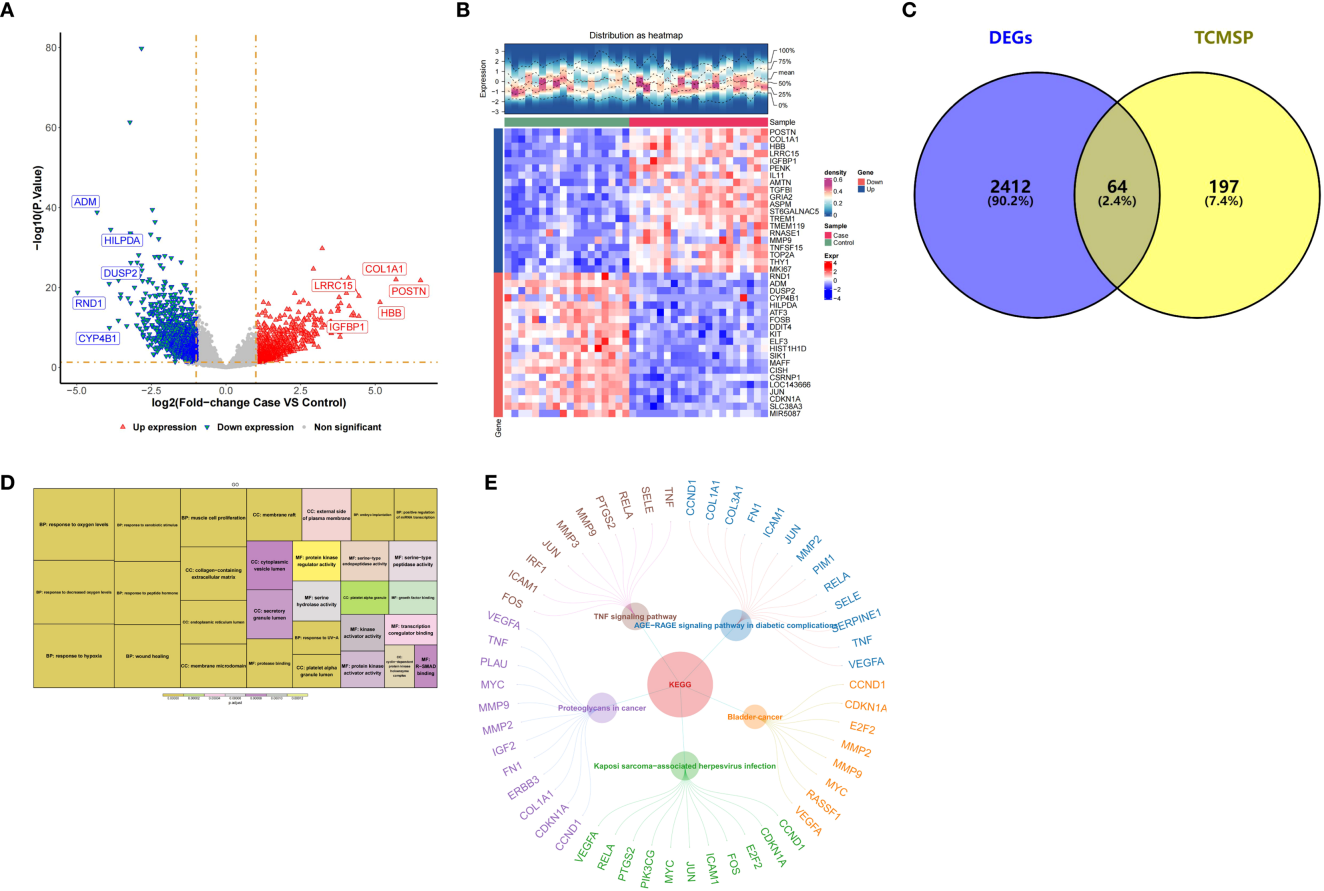
**(A)** Volcano plot of differentially expressed genes in the GSE114007 dataset, showing the log-fold change and adjusted P-values of genes; **(B)** Heatmap of differentially expressed genes, intuitively reflecting the expression levels of genes in different samples; **(C)** Venn diagram showing the intersection of differentially expressed genes and the drug targets of Bi Pain Mixture obtained 64 candidate genes. **(D)** The GO enrichment analysis results for candidate genes, displaying the enriched cellular components, molecular functions, and biological processes related entries. **(E)** The KEGG enrichment analysis results of candidate genes, displaying the relevant signaling pathways.

Through the identification of candidate genes targeted by BM and functional enrichment analysis, we obtained preliminary insights into their biological relevance to OA. In GSE114007, 2,476 DEGs were identified, comprising 1,329 upregulated and 1,147 downregulated genes (**Figures 2A, B**), which overlapped with 261 drug targets of BM, further identifying 64 candidate genes (**Figure 2C**). These candidates were collectively enriched into 1,347 GO terms (55 cellular components (CCs), 112 molecular functions (MFs), and 1,180 biological processes (BPs)), primarily involving functions such as response to oxygen levels, response to decreased oxygen levels, among others (**Figure 2D**). Additionally, 116 signaling pathways were excavated for candidate genes, such as the TNF signaling pathway, the AGE−RAGE signaling pathway in diabetic complications, and so on (**Figure 2E**).

### 3.3 Protein interaction network identifies 7 candidate key genes (human: GSE114007)

**Figure 3:**
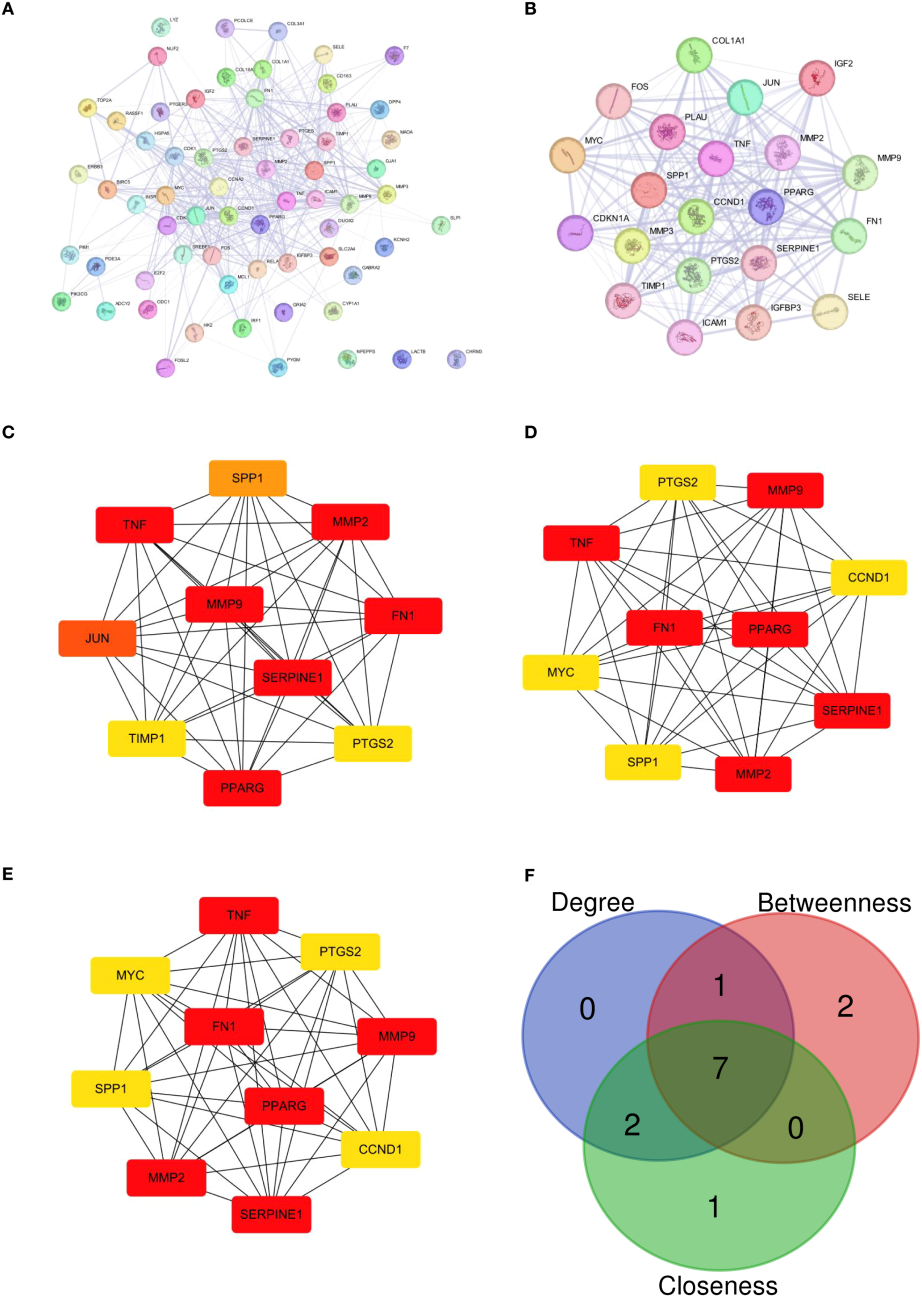
**(A)** Schematic diagram of the interaction between some genes in the PPI network, showing the interaction between top2 and genes such as BIRC, NUF2, and RASSF1. **(B)** Gene clusters identified by the MCODE plugin with the highest connectivity. **(C–E)** Degree, Betweenness, Closeness Algorithm Analysis Results. **(F)** The intersection of results from 3 algorithms yields 7 candidate key genes.

Building upon the identified candidate genes, we performed a PPI network analysis to elucidate their interconnectivity and prioritize central regulators within the OA pathological network. In the PPI network, 60 candidate genes formed 640 regulatory relationships. For example, top2 interacted at the protein level with multiple genes such as BIRC, NUF2, and RASSF1 (**Figure 3A**). Subsequently, the top 1 connectivity gene cluster identified by the MCODE plugin (**Figure 3B**) was further analyzed using Degree, Betweenness, and Closeness algorithms (**Figures 3C–E**). The intersection of genes identified by these algorithms yielded 7 candidate key genes, namely MMP9, MMP2, SPP1, PTGS2, SERPINE1, TNF, and FN1 (**Figure 3F**).

### 3.4 Machine learning identifies MMP9, MMP2, and SPP1 as potential biomarkers (human: GSE114007, GSE169077)

**Figure 4:**
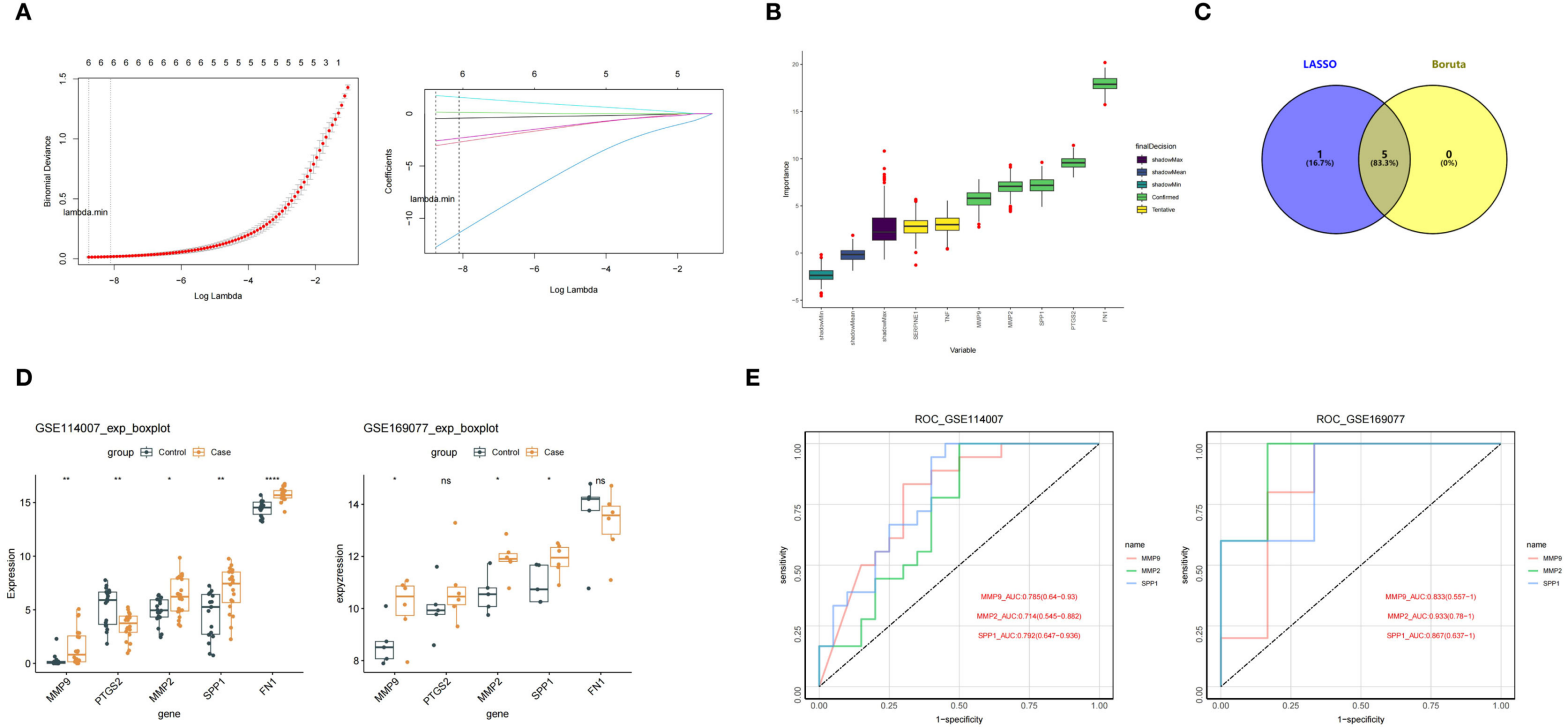
**(A)** LASSO regression analysis results show that the 6 feature genes identified when the error is minimal. **(B)** Boruta analysis results, displaying the 5 extracted feature genes. **(C)** Venn diagram, presenting the intersection of LASSO regression and Boruta analysis results yields 5 key feature genes. **(D)** MMP9, MMP2, and SPP1 expression levels comparison between case and control groups in the GSE114007 and GSE169077 datasets. **(E)** MMP9, MMP2, and SPP1 in the two datasets, showing their diagnostic performance.

To distill the diagnostic relevance of the candidate key genes, we conducted machine learning-based screening for potential biomarkers. In LASSO regression analysis, 6 feature genes 1 (MMP9, MMP2, SPP1, PTGS2, SERPINE1, and FN1) were identified when the error was minimized (lambda.min = 0) (**Figure 4A**). Simultaneously, 5 feature genes 2 (MMP9, MMP2, SPP1, PTGS2, and FN1) were also mined in Boruta analysis (**Figure 4B**). The intersection of these feature genes was taken to identify 5 key feature genes (MMP9, MMP2, SPP1, PTGS2, and FN1) (**Figure 4C**). Among them, MMP9, MMP2, and SPP1 were considered potential biomarkers for OA as they were significantly upregulated in case samples of both GSE114007 and GSE169077 (**Figure 4D**). Subsequently, these potential biomarkers exhibited certain diagnostic performance for OA, with their area under curve (AUC) values for ROC curves exceeding 0.7 in both datasets (**Figure 4E**).

### 3.5 Diagnostic nomogram based on potential biomarkers (human: GSE114007)

**Figure 5:**
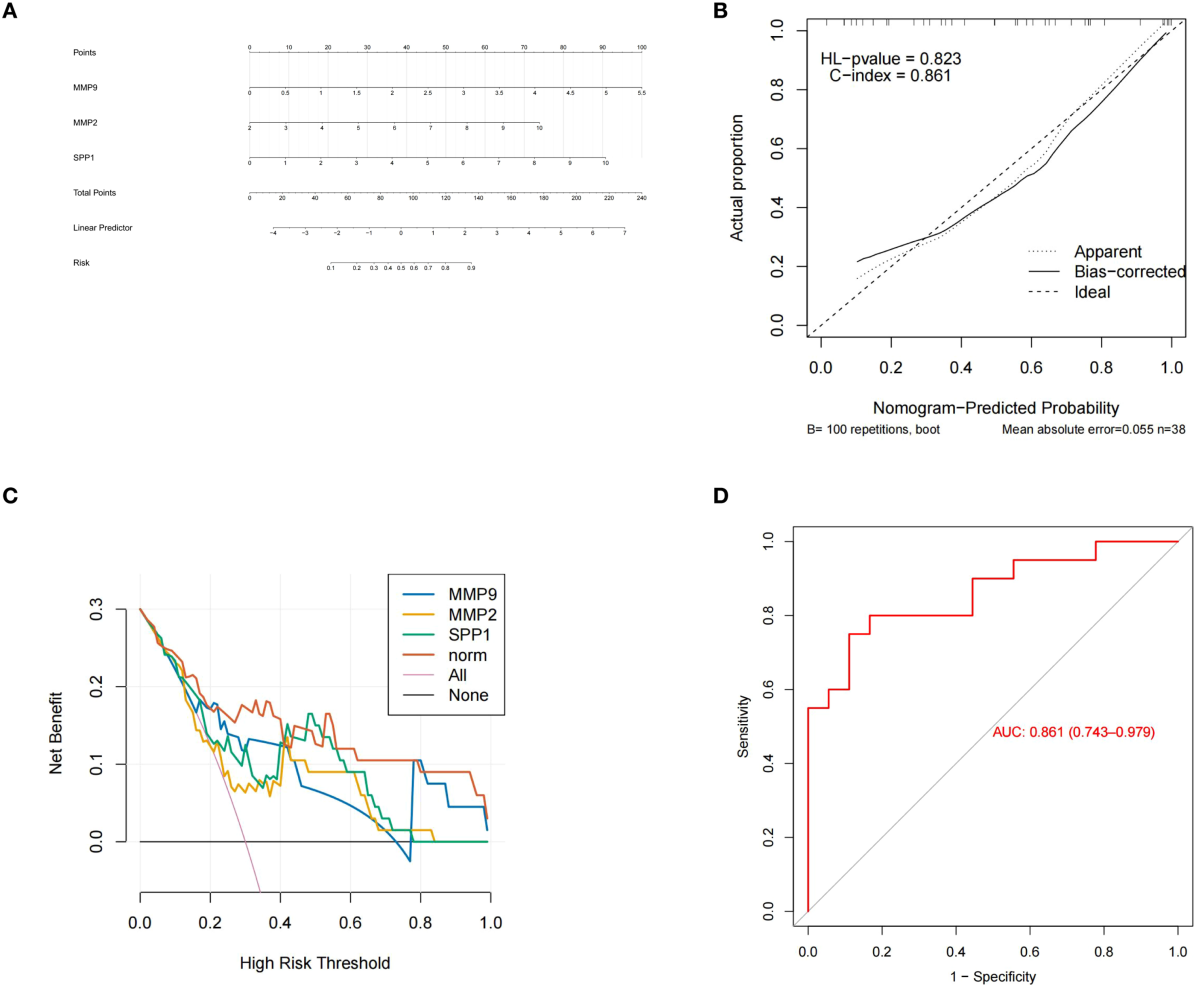
**(A)** Potential biomarker nomogram, showing the score axes corresponding to the three potential biomarkers MMP9, MMP2 and SPP1, as well as the relationship between the total score and the probability of osteoarthritis occurrence; **(B)** Calibration curve, showing that the C index is 0.861; **(C)** The DCA analysis results indicated that the net benefit of the nomogram model was greater than that of a single biomarker; **(D)** The ROC curve shows that the AUC value is 0.861.

To preliminarily assess the integrated diagnostic potential of MMP9, MMP2, and SPP1 for OA, we constructed an exploratory nomogram based on the GSE114007 dataset (**Figure 5A**). Afterwards, its predictive performance was further evaluated. The calibration curve showed C-index of 0.861, which exceeded 0.7 (**Figure 5B**). In DCA analysis, the net benefit of the nomogram was greater than that of individual potential biomarkers (**Figure 5C**). Moreover, the AUC value of the ROC curve was 0.861, also exceeding 0.7 (**Figure 5D**). These results consistently indicated that the nomogram of potential biomarkers had certain predictive performance for OA. However, as this nomogram is an exploratory tool constructed solely on transcriptomic data, its clinical applicability requires further validation in future prospective cohorts that incorporate clinical variables.

### 3.6 Signaling pathways and gene networks associated with potential biomarkers (human: GSE114007)

**Figure 6:**
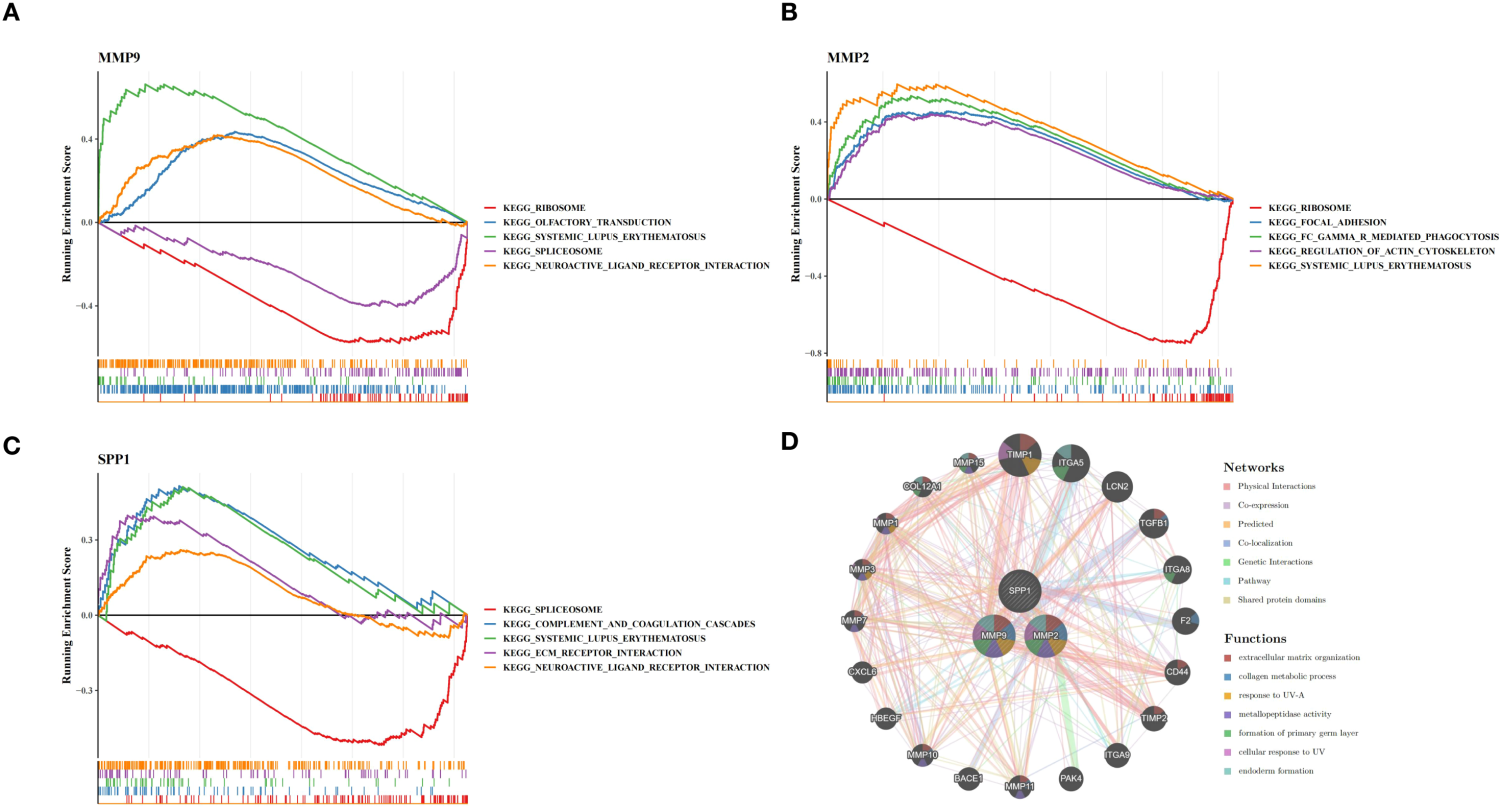
**(A–C)** GSEA analysis showed that potential biomarkers were jointly involved in the signaling pathways of systemic lupus erythematosus; MMP9 and MMP2 are enriched in the ribosomal signaling pathway; MMP9 and SPP1 are enriched in the spliceosome and neuroactive ligand-receptor interaction signaling pathways. **(D)** The top 20 genes and 7 signaling pathways related to potential biomarkers in the GGI network.

Elucidating the broader functional context of these potential biomarkers, we performed pathway and network enrichment analyses. GSEA was employed to further provide insight into the function of potential biomarkers. The results indicated that all biomarkers were collectively involved in systemic lupus erythematosus. Additionally, MMP9 and MMP2 were enriched together in the ribosome pathway. MMP9 and SPP1 were significantly enriched in the spliceosome and neuroactive ligand receptor interaction pathways (**Figures 6A–C**). Subsequently, the top 20 genes associated with the potential biomarkers (e.g., TIMP1, ITGA5) and seven signaling pathways were identified in the GGI network. These pathways included extracellular matrix organization, collagen metabolic process, and others (**Figure 6D**).

### 3.7 Altered immune infiltration in OA and correlation with potential biomarkers (human: GSE114007)

**Figure 7:**
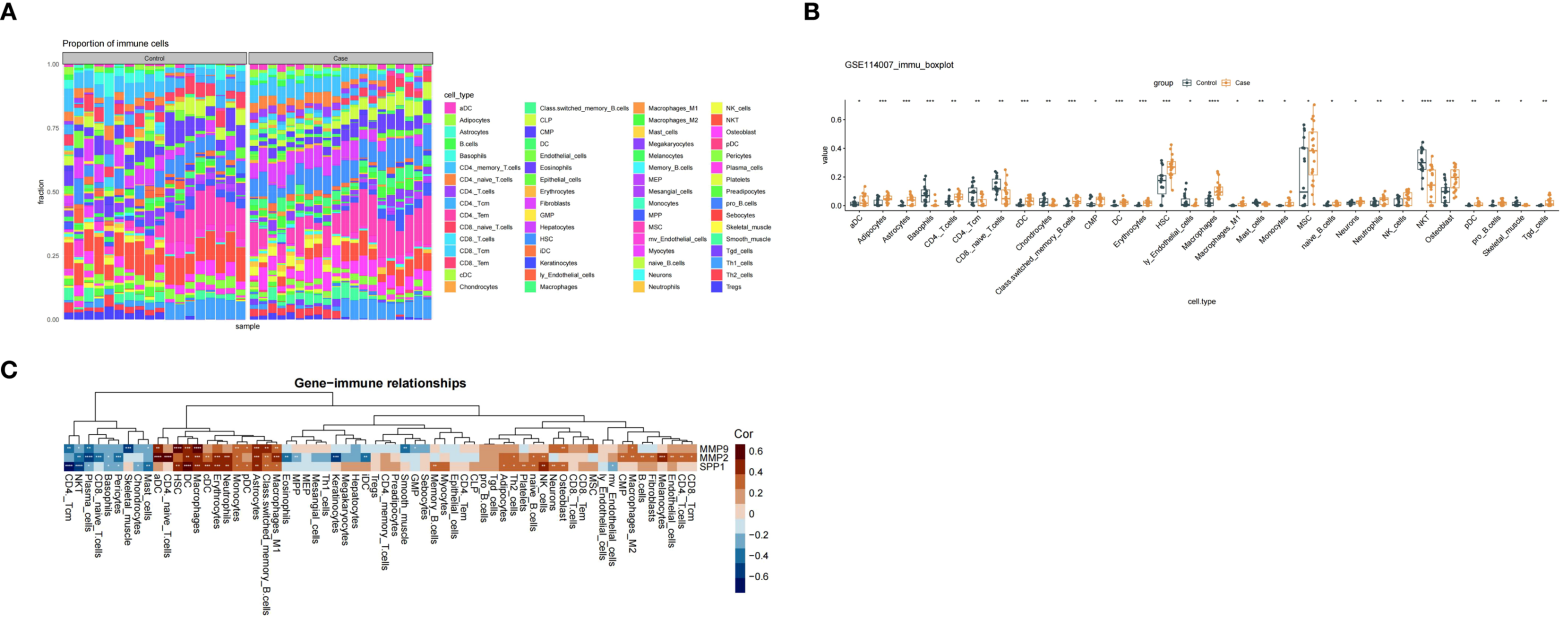
**(A)** The results of xcell analysis on the infiltration of 64 types of immune cells are presented; **(B)** Comparison of 22 immune cells with higher infiltration scores and 8 immune cells with lower infiltration scores; **(C)** In the correlation analysis of potential biomarkers and 64 types of immune cells, the correlations between MMP2 and aDC, and SPP1 and CD4_Tcm were displayed.

To elucidate the immunological context in which these potential biomarkers operate, we assessed immune cell infiltration patterns in OA. In this study, the infiltration of 64 immune cells was explored according to xcell (**Figure 7A**), of which, 22 immune cells, such as adipocytes, astrocytes, had significantly higher infiltration scores in case samples, whereas the remaining 8 immune cells (e.g., basophils, mast cells), were the opposite (**Figure 7B**). Furthermore, in the correlation analysis of potential biomarkers with 64 immune cells, MMP2 showed the largest positive correlation with aDC (cor=0.66, p ≤ 0.0001), while SPP1 showed the largest negative correlation with CD4 Tcm (cor = -0.75, p ≤ 0.0001) (**Figure 7C**).

### 3.8 Regulatory networks and molecular docking predict interactions between BM compounds and potential biomarkers (database prediction)

**Figure 8:**
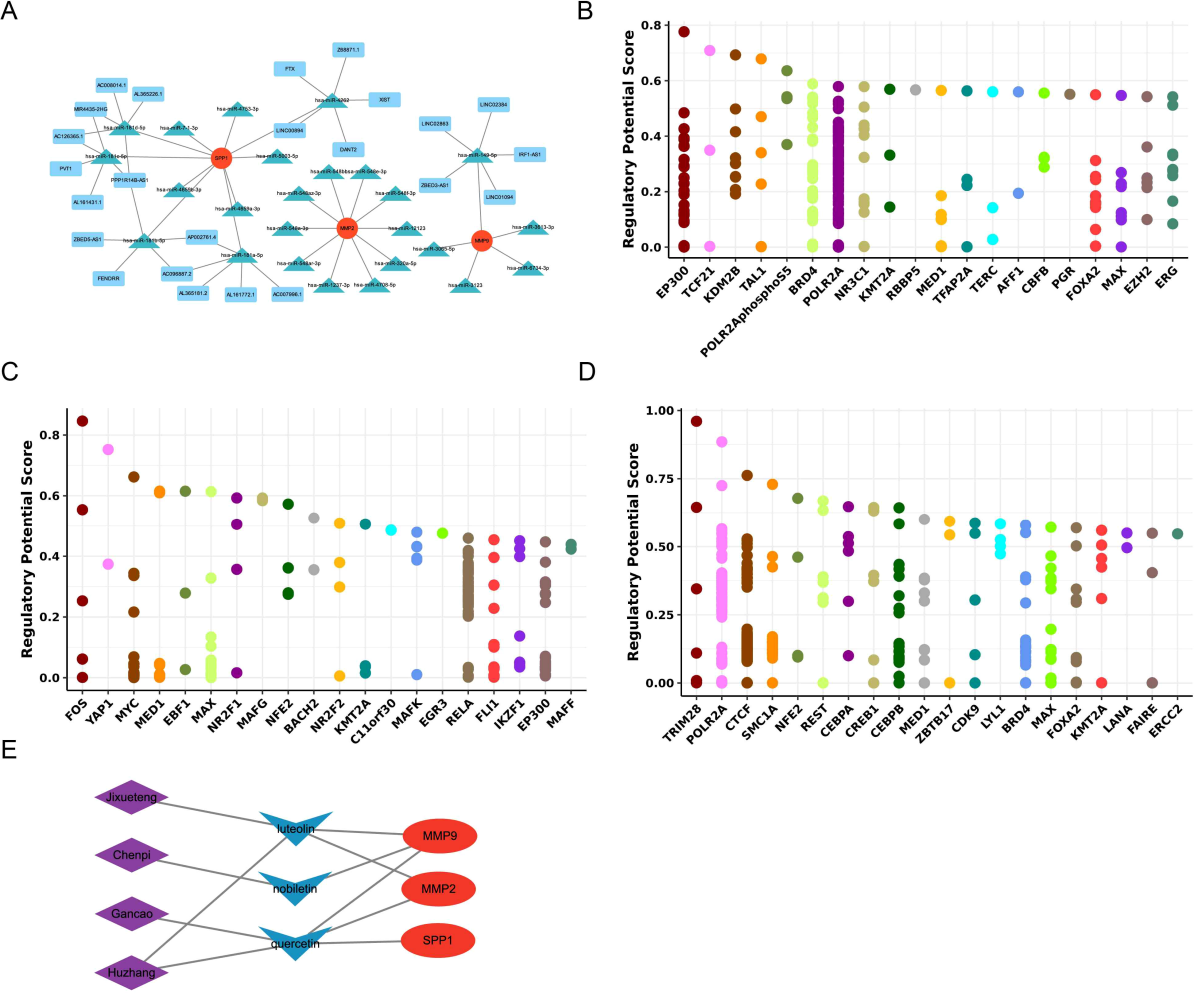
**(A)** The lncRNA-miRNA-mRNA network shows the regulatory relationships among 25 mirnas, 24 lncrnas and 3 potential biomarkers; **(B–D)** The prediction results of the key transcription factors of MMP9, MMP2 and SPP1; **(E)** The figure shows the herb - active compound - gene network, presenting the pairs of relationships among four herbs, three active components and potential biomarkers.

Building upon these potential biomarkers, we explored their upstream regulatory networks and performed molecular docking to predict their mechanistic roles in OA. In the lncRNA-miRNA-mRNA network of potential biomarkers, 25 miRNAs, 24 lncRNAs, and 3 potential biomarkers were included. They formed regulatory relationships included PVT1 - has-miR-181c-5p - SPP1, LINC02863 - has-miR-149-5Pp - MMP9 and so on (**Figure 8A**). After that, TFs corresponding to potential biomarkers were also predicted, and the results showed that FOS, EP300, and TRIM28 were the most critical TFs for MMP9, MMP2, and SPP1, respectively (**Figures 8B–D**). Furthermore, 4 herbal (Jixueteng, Chenpi, Gancao, and Huzhang), and 3 active ingredients (luteolin, quercetin, and nobiletin) were used to construct the herbal-active compounds-gene network with potential biomarkers. The relationship pairs formed included Jixueteng-luteolin-MMP9, Huzhang-quercetin-SPP1, and so on (**Figure 8E**). Finally, these active compounds were applied for molecular docking with potential biomarkers to predict the binding affinity, in which luteolin demonstrated the most favorable docking effect with MMP9 in the simulation, and its predicted docking binding energy was -10.1 kcal/mol (**Supplementary Figure 2**).

### 3.9 Single-cell profiling of OA cartilage identifies key cell types (human: GSE255460)

**Figure 9:**
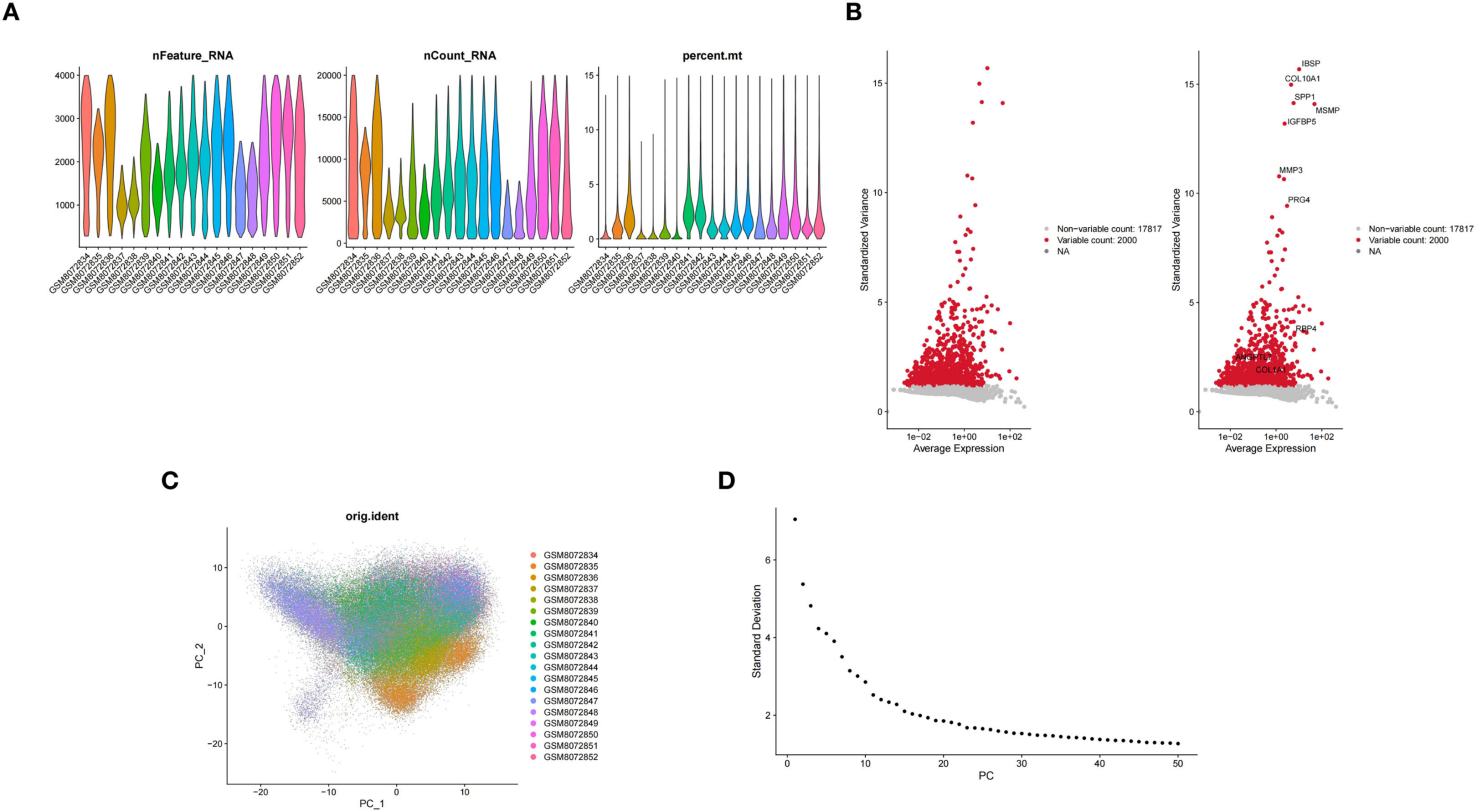
**(A)** Violin plot, showing the distribution of nFeature RNA, nCount RNA and percent.mt in the samples after quality control; **(B)** Display diagram of highly variable genes; **(C)** Principal Component analysis (PCA) plot, showing the heterogeneity between cells; **(D)** The analysis graph of the integrated data shows that the cell distribution is uniform without obvious outliers.

**Figure 10:**
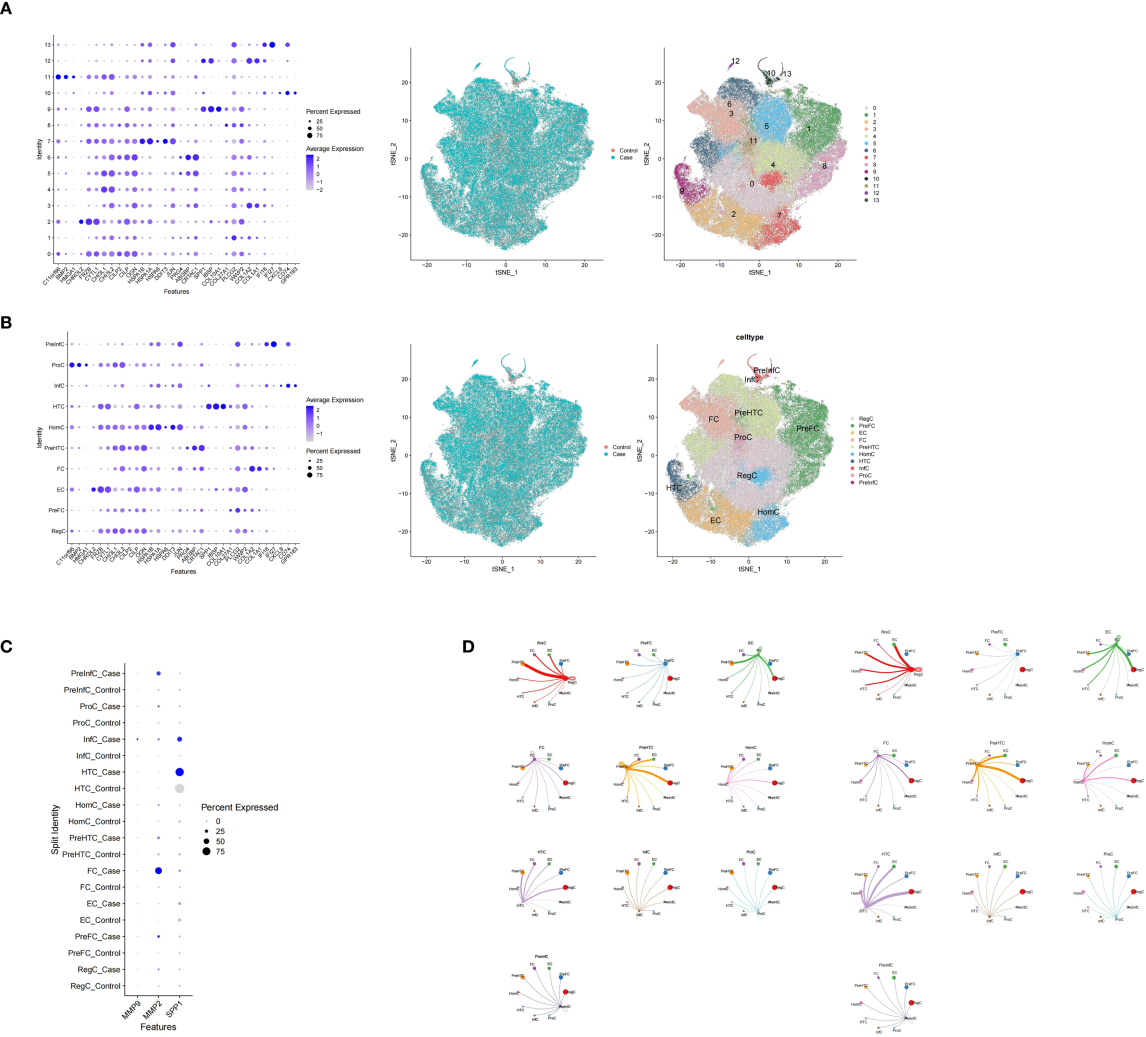
**(A)** The T-distribution random neighborhood embedding (tSNE) clustering analysis diagram identified 14 cell clusters; **(B)** The results of cell annotation determined 10 types of cells; **(C)** The distribution of potential biomarkers in different cell types; **(D)** The results of intercellular communication analysis show the communication changes among different cell types.

We next used scRNA-seq to resolve the cellular expression patterns of these potential biomarkers and the associated changes in the joint communication network during OA. Moving on to the single-cell RNA sequencing analysis, the distribution of nFeature RNA (the number of genes detected per cell), nCount RNA (the total gene expression per cell), and percent.mt (the proportion of mitochondrial genes per cell) in the quality-controlled samples was visualized using violin plots (**Figure 9A**). In addition, highly variable genes within the GSE255460 dataset were identified and displayed (**Figure 9B**). To explore the heterogeneity among cells, PCA was conducted (**Figure 9C**), while the integrated data indicated that cells were evenly mixed without significant outliers. Subsequently, the top 30 principal components were selected for further analysis (**Figure 9D**). Before cell annotation, TSNE clustering analysis was performed, identifying a total of 14 cell clusters (**Figure 10A**). The subsequent cell annotation identified 10 cell types: regulatory chondrocytes (RegC), proliferative chondrocytes (ProC), homeostatic chondrocytes (HomC), prehypertrophic chondrocytes (preHTC), effector chondrocytes (EC), fibrochondrocytes (FC), hypertrophic chondrocytes (HTC), pre-fibrochondrocytes (preFC), pre-inflammatory chondrocytes (preInfC), and inflammatory chondrocytes (InfC) (**Figure 10B**). Annotation results revealed that the SPP1 gene is highly expressed in HTC and InfC cells, MMP2 is abundantly expressed in FC cells, and MMP9 is relatively enriched in InfC cells. Thus, HTC, InfC, and FC were selected as key cell types, suggesting that these cells may play significant roles in the drug effects on OA (**Figure 10C**). Moreover, the analysis of cell-cell interactions revealed that in the control group, HTC cells played a key role in communication with other cells. However, in osteoarthritis, their interactions weakened, except with preHTC and FC cells, where communication remained stable. Meanwhile, IfnC cells showed no significant changes between the groups. In contrast, FC cells had stronger communication with preHTC cells and weaker interactions with EC and RegC cells in osteoarthritis, suggesting that preHTC cells may be crucial in the progression of the disease (**Figure 10D**). In contrast, the interaction patterns of InfC cells did not change notably between the control and disease groups. Additionally, the interactions between FC and preHTC cells were strengthened in the disease group, whereas their interactions with EC and RegC were weakened. These findings suggest that pre-HTC cells may also play a key role in the progression of osteoarthritis.

### 3.10 BM downregulates potential biomarker expression (rat model)

**Figure 11:**
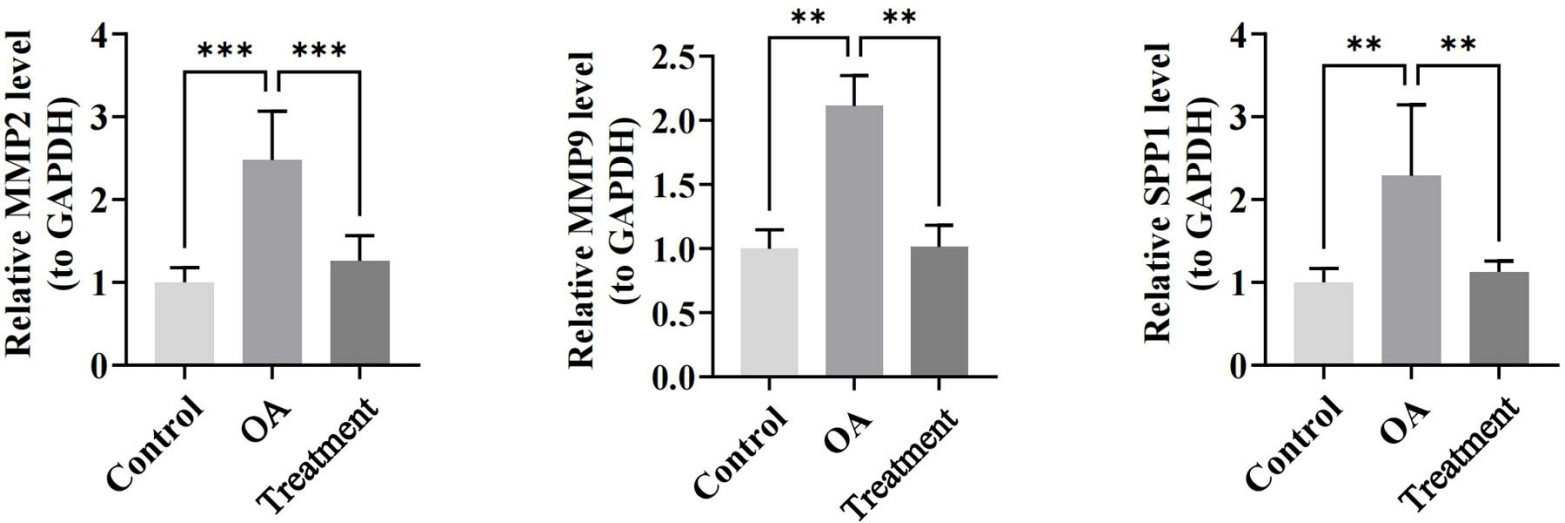
It is a bar chart of the relative expression levels of MMP2, MMP9, and SPP1 in the control group,OA group, and treatment group. The expression in the OA group is significantly higher thanthat in the control group, and the expression in the treatment group is significantly lower than that in the OA group and close to the control group.**: p < 0.01, ***: < 0.001.

RT-qPCR analysis was subsequently performed in the rat model to assess the expression of these biomarkers under BM intervention. Compared to the control group, the expression of MMP9, MMP2, and SPP1 in OA group was significantly upregulated (P < 0.01). Their expression trends were consistent with those in GSE114007 and GSE169077 datasets, revealing the reliability of aforementioned results. Besides, compared to the OA group, MMP9, MMP2, and SPP1 were significantly downregulated in the treatment group (P < 0.01). Notably, the expression level in the treatment group was closer to control group, implying that BM might have a corrective or normalizing effect on the pathological processes associated with OA (**Figure 11**).

## 4 Discussion

OA is a degenerative joint disease characterized by cartilage degradation, synovial inflammation, osteophyte formation, and pain. Current therapies focus on symptom management, but targeted interventions remain limited (42). The Bitong mixture (BM), a traditional herbal formula, has shown empirical efficacy in alleviating OA symptoms, though its molecular mechanisms are poorly understood (16–18). This study systematically explored the potential mechanisms and biological markers of BM in treating OA by integrating bioinformatics, animal experiments, and computational simulations. First, MMP9, MMP2, and SPP1 were screened as BM-related candidate markers based on human transcriptome data; subsequently, a rat OA model was constructed to preliminarily verify that BM could alleviate joint pathology and downregulate the expression of biological markers, transitioning from correlation analysis based on human data to validation through animal experiments. Network pharmacology and molecular docking suggested that the active components of BM (such as luteolin) may directly act on these targets. Single-cell analysis further revealed that these markers are primarily enriched in hypertrophic and inflammatory chondrocyte subpopulations, indicating that BM may influence matrix metabolism and the immune microenvironment by regulating these cell functions. In summary, this study suggests that BM may alleviate the disease progression of OA by regulating MMP9, MMP2, and SPP1 through a synergistic effect of multiple components, thereby affecting extracellular matrix metabolism and the immune microenvironment. These results provide preliminary evidence and direction for further elucidating its mechanisms of action and exploring potential clinical applications.

### 4.1 The role and mechanism of potential biomarkers in osteoarthritis

Matrix metalloproteinase 9 (MMP9) belongs to the matrix metalloproteinase family. Its main function is to degrade extracellular matrix components such as collagen and gelatin (43).Beyond ECM remodeling, MMP9 participates in inflammatory cascades by facilitating leukocyte infiltration and amplifying pro-inflammatory cytokine release (e.g., IL-1β, TNF-α), which perpetuates synovial inflammation and joint damage (44). Under inflammatory stimulation, the expression of MMP9 will be induced to increase, forming a vicious cycle and exacerbating the development of osteoarthritis (45). In addition, MMP9 can regulate osteogenesis/osteolysis by inhibiting bone resorption and promoting bone formation (46). In osteoarthritis, the expression of MMP9 is significantly upregulated (45), and its expression level is positively correlated with the severity of the disease. This indicates that MMP9 plays a key role in the pathogenesis of osteoarthritis and can be used as an important indicator for evaluating the condition of osteoarthritis (47, 48). Its potential mechanisms may involve degrading articular cartilage, promoting inflammatory responses and the release of inflammatory mediators, while playing a significant role in osteophyte formation.

Matrix metalloproteinase 2 (MMP2), a crucial member of the MMP family, degrades extracellular matrix components including type IV collagen and gelatin (49, 50). In OA, MMP2 expression is markedly upregulated from early disease stages, with levels escalating in correlation with radiographic and histological severity scores (e.g., OARSI grading). Studies demonstrate its capacity to degrade cartilage and synovial ECM, disrupting joint integrity and function. This elevation from early OA stages suggests MMP2’s promotive role in disease progression, highlighting its potential as a potential diagnostic biomarker and therapeutic target (51–53). Moreover, MMP2 regulates cellular proliferation, migration, and differentiation, potentially mediating OA pathology by modulating chondrocyte and synoviocyte biological behaviors.

Secreted phosphoprotein 1 (SPP1/osteopontin), a multifunctional extracellular matrix phosphoglycoprotein involved in cell adhesion, migration, and signaling, shows significant upregulation in OA (54). Research reveals SPP1 enhances inflammatory cell adhesion/migration, amplifies inflammatory responses, and modulates chondrocyte proliferation/differentiation while suppressing cartilage matrix synthesis. Elevated SPP1 levels in OA patients’ articular cartilage and synovial tissue correlate strongly with inflammatory severity and cartilage degeneration, indicating its pivotal role in OA pathogenesis through inflammation regulation and chondrocyte functional modulation (55). Additionally, SPP1’s involvement in bone metabolism suggests its potential contribution to osteophyte formation and joint deformity via bone remodeling alterations in OA.

Notably, the upregulation of these potential biomarkers in OA patients and their normalization following BM intervention suggest that BM may inhibit MMP activity and osteopontin-mediated pathological calcification, thereby preserving cartilage integrity (56). These potential biomarkers show preliminary diagnostic efficacy (AUC > 0.7) in different data sets, suggesting they have good diagnostic potential, and future efforts can focus on further validating their clinical application value. The discovery of these potential biomarkers not only serves as an early assessment indicator with potential value in OA diagnosis but also provides a theoretical basis for developing new treatment strategies based on BM.

### 4.2 Pathway analysis reveals key BM-regulated mechanisms

The GSEA results demonstrated that all three potential biomarkers are collectively involved in systemic lupus erythematosus (SLE), suggesting autoimmune involvement in OA pathogenesis. In OA patients, synovial immune dysregulation resembling SLE may trigger inflammatory cytokine overproduction (e.g., IL-6, TNF-α) and cartilage breakdown. Autoimmune components may promote synovial immune cell infiltration, accelerating joint destruction through cytokine-mediated pathways (57, 58), highlighting immunomodulation as a potential OA therapeutic target.

MMP9 and MMP2 co-enrich in the ribosome pathway, a central protein synthesis site crucial for cellular homeostasis. This enrichment implies dual regulatory mechanisms in osteoarthritis: 1) MMP9/MMP2 may modulate ribosomal biogenesis and function, potentially influencing ribosome assembly or mRNA translation efficiency; 2) Their expression could conversely depend on ribosomal signaling. In OA chondrocytes, ribosomal dysfunction may impair synthesis of cartilage matrix proteins like collagen, reducing tissue elasticity and accelerating degradation through compromised structural integrity (59, 60).

MMP9 and SPP1 exhibited dual-pathway enrichment in spliceosome function and neuroactive ligand-receptor interactions. The spliceosome pathway enrichment suggests their potential regulatory roles in mRNA splicing, where aberrant processes in OA could lead to chondrocyte dysfunction through impaired metabolic/proliferative/differentiation pathways (61, 62). Concurrently, their neuroactive ligand-receptor association implies possible involvement in pain modulation, particularly through regulating OA-elevated nociceptive mediators like substance P and CGRP (63, 64). These findings propose that MMP9/SPP1 may mediate OA progression through mRNA processing accuracy and pain signal transduction, offering potential therapeutic targets for both cartilage degeneration and pain management in OA (65).

In summary, the signaling pathways associated with these potential biomarkers encompass immune regulation, protein synthesis, gene expression regulation, and neural signal transduction at multiple levels. This not only provides a new perspective for understanding the pathogenic mechanism of OA but also offers potential clues for the mechanistic research of BM in treating OA.

### 4.3 Association analysis of immune cells and potential biomarkers

Using xCell, we conducted immune cell infiltration analysis on the GSE114007 dataset to systematically profile 64 immune cell types in OA pathogenesis. Significant infiltration differences emerged between OA cases and controls, with 22 cell types—including adipocytes and astrocytes—exhibiting higher infiltration scores in OA samples. Enhanced adipocyte infiltration may modify inflammatory microenvironments through fat-derived mediators like leptin (pro-inflammatory) and adiponectin, which regulate immune responses (66). For astrocytes, their increased presence suggests neuro-immune crosstalk in OA, potentially mediated by neurotransmitters and cytokines influencing immune cell activity and inflammation progression (67).

The infiltration scores of eight immune cells, including basophils and mast cells, were significantly lower in OA samples than in controls. Basophils and mast cells regulate allergic responses and inflammation by releasing histamine, leukotrienes, and other mediators (68). Their reduced infiltration in OA may impair inflammation regulation, exacerbating uncontrolled inflammatory responses and disease progression.

To explore potential relationships between potential biomarkers and immune cells, Spearman correlation analysis was conducted. Results revealed the strongest positive correlation between MMP2 and activated dendritic cells (aDCs) (cor = 0.66, P ≤ 0.0001). As crucial antigen-presenting cells, aDCs process antigens and activate T cells to initiate immune responses. The MMP2-aDC correlation suggests MMP2 may interact with aDCs to modulate immune regulation and inflammatory responses in osteoarthritis pathogenesis. Functionally, MMP2 degrades extracellular matrix components, potentially releasing cartilage breakdown products that activate aDCs. Conversely, cytokines secreted by activated aDCs might upregulate MMP2 expression, forming a positive feedback loop that exacerbates inflammation and joint damage (69–71).

SPP1 exhibited the strongest negative correlation with CD4_Tcm (central memory CD4+ T cells) (cor = -0.75, P ≤ 0.0001). CD4_Tcm plays crucial roles in maintaining immune memory and regulating immune responses (72). The strong negative correlation suggests that SPP1 might impair immune memory maintenance and immune balance through suppressing CD4_Tcm functions, potentially driving OA pathogenesis. The encoded osteopontin (OPN) interacts with multiple cell surface receptors to regulate cell adhesion, migration, and signaling (73, 74). In summary, through immune infiltration analysis, we speculate that BM may regulate potential biomarkers to inhibit the excessive activation of dendritic cells and reduce the functional suppression of CD4+ central memory T cells, alleviating the vicious cycle of inflammation and tissue destruction in osteoarthritis, which helps restore local immune homeostasis in the joints and ultimately exerts a therapeutic effect in alleviating the progression of OA. However, the specific related effects still need further verification through subsequent experiments (75, 76).

### 4.4 Analysis of molecular docking results and mechanisms

In molecular docking analysis, we constructed an herb-active compound-gene network linking potential biomarkers and herbal active compounds to investigate their interaction mechanisms. The network identified four herbs (Jixueteng, Chenpi, Gancao, and Huzhang) and three active compounds (luteolin, quercetin, nobiletin) associated with the potential biomarkers.

Among them, luteolin demonstrated the strongest binding affinity with MMP9 at −10.1 kcal/mol. Binding energy (negative values indicate stability) revealed their high-affinity interaction and capability to form a stable complex. The excellent binding affinity between luteolin and MMP9 underscores luteolin’s potential as a direct MMP9 inhibitor. Studies have shown that luteolin exhibits multiple biological activities including anti-inflammatory, antioxidant, and anti-tumor effects (77–79). In osteoarthritis treatment, luteolin may competitively block MMP9’s catalytic site, thereby reducing collagenase activity and cartilage degradation (80). This aligns with BM’s observed efficacy in reversing OA potential biomarker expression toward normal levels.

Additionally, luteolin may exert therapeutic effects on osteoarthritis by modulating other signaling pathways. Studies indicate that luteolin inhibits nuclear factor-κB (NF-κB) pathway activation, reducing the expression and release of inflammatory factors (81–83). In osteoarthritis, inflammatory response significantly contributes to joint damage. NF-κB pathway activation promotes production of inflammatory cytokines like iIL-1β and TNF-α, exacerbating joint inflammation and cartilage degradation (84). By suppressing the NF-κB pathway, luteolin likely alleviates inflammatory responses, thereby demonstrating therapeutic potential for osteoarthritis.

Studies indicate that luteolin promotes chondrocyte proliferation and differentiation while inhibiting apoptosis. In osteoarthritis, chondrocyte damage and apoptosis reduce cartilage matrix synthesis. By enhancing chondrocyte proliferation and differentiation, luteolin may increase cartilage matrix production, aiding the repair of damaged articular cartilage (85, 86).

In summary, molecular docking simulation shows that there is a potential high-affinity binding pattern between luteolin and MMP9, suggesting that it may play a potential role in alleviating extracellular matrix degradation, regulating inflammatory response, and promoting cartilage homeostasis by modulating MMP9 activity and downstream related signaling pathways. This provides a preliminary theoretical basis for the hypothesis of “BM regulating the improvement of OA pathological process through potential biomarkers” in this study, and future exploration of the mechanism of action of BM and the development of new drugs can be based on this. However, since molecular docking analysis is only a predictive study, its results need to be further validated through functional experiments.

### 4.5 Conclusion and limitations

This study first systematically explored the possible mechanisms of BM in OA by integrating multi-omics analysis and experimental validation. The results showed that BM may affect the OA process and produce therapeutic effects by regulating extracellular matrix remodeling and immune microenvironment modulation mediated by MMP9, MMP2, and SPP1, providing references and new insights for understanding the potential molecular mechanisms of traditional Chinese medicine compound treatment for OA. Although this study integrates bioinformatics and animal experiments, there are still the following limitations: (1) The sample size, heterogeneity, and late-stage OA single-cell data of public datasets limit the understanding of the disease as a whole, and there is a risk of model overfitting; (2) The MIA model has the defect of not being able to fully simulate the complex disease course of human OA, and currently lacks solvent and positive drug controls; (3) The findings of this study are mainly based on computational predictions and mRNA levels, lacking direct evidence, protein levels, and functional experimental validation in OA patients. Based on this, future research should deepen in the following directions: (1) Conduct larger-scale prospective clinical studies to systematically collect a series of samples from OA patients receiving BM treatment, and validate the function of markers at the species level; (2) Supplement solvent and positive drug controls, and validate the role of markers and key interaction mechanisms at the protein and functional levels through experiments such as Western blotting, immunohistochemistry, and gene knockdown/overexpression; (3) Utilize technologies such as organoids and spatial transcriptomics to elucidate the regulatory network of BM on chondrocyte subpopulations; (4) Integrate computational and experimental approaches to clarify the compound material basis and optimize its design, ultimately achieving the transformation towards evidence-based precision treatment.

## Data Availability

The datasets presented in this study can be found in online repositories. The names of the repository/repositories and accession number(s) can be found in the article/**Supplementary Material**.

## Supplementary material

Supplementary Figure 1 Schematic overview illustrating the integrated study design for investigating BM in OA.

Supplementary Figure 2 The figure shows the visual display of the docking result between luteolin and MMP9 molecules, indicating that the docking binding energy is -10.1 kcal/mol. It should be noted that this analysis is predictive and has not been functionally validated; therefore, these findings are hypothesis-generating and intended only for guidance in future mechanistic research.

Supplementary Table 1 Summary of the GEO datasets used for bioinformatics analysis, including dataset accession numbers, sample labels, disease status, species, tissue source, and technology platform.
