# Supplementary material for: Identification of potential biomarkers related to the Bitong mixture in osteoarthritis based on bioinformatics and network pharmacology, and exploration of the mechanism involved
Source: Front Immunol. 2026 Feb 13;17:1739355. doi: 10.3389/fimmu.2026.1739355 (PMC12946070; doi:10.3389/fimmu.2026.1739355)
Supplement: Supplementary Figure 1 — Schematic overview illustrating the integrated study design for investigating BM in OA. [file DataSheet1.docx]

Supplementary Material

# Supplementary Figures and Tables

## Supplementary Figures


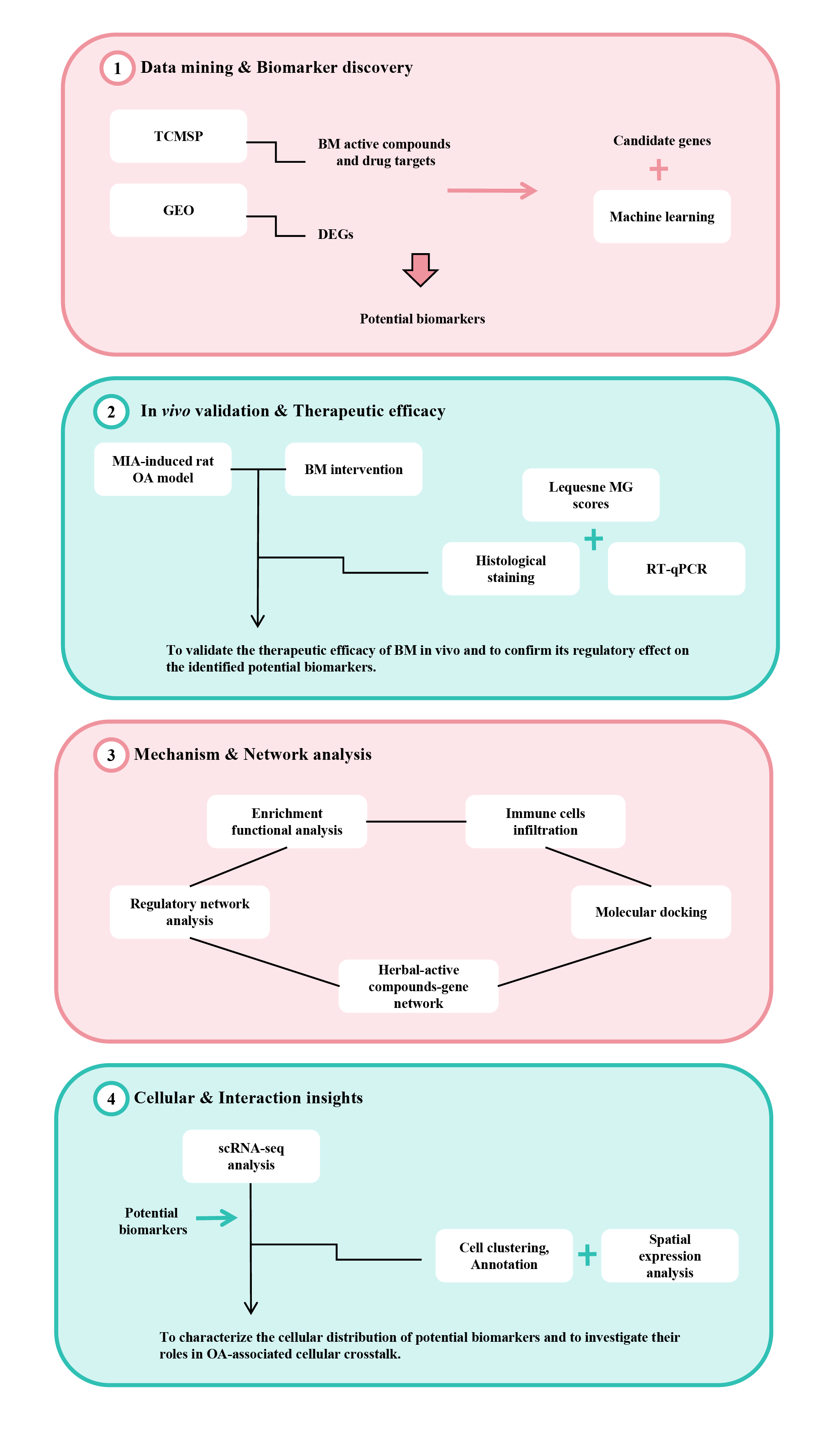


**Supplementary Figure 1.** Schematic overview illustrating the integrated study design for investigating BM in OA.


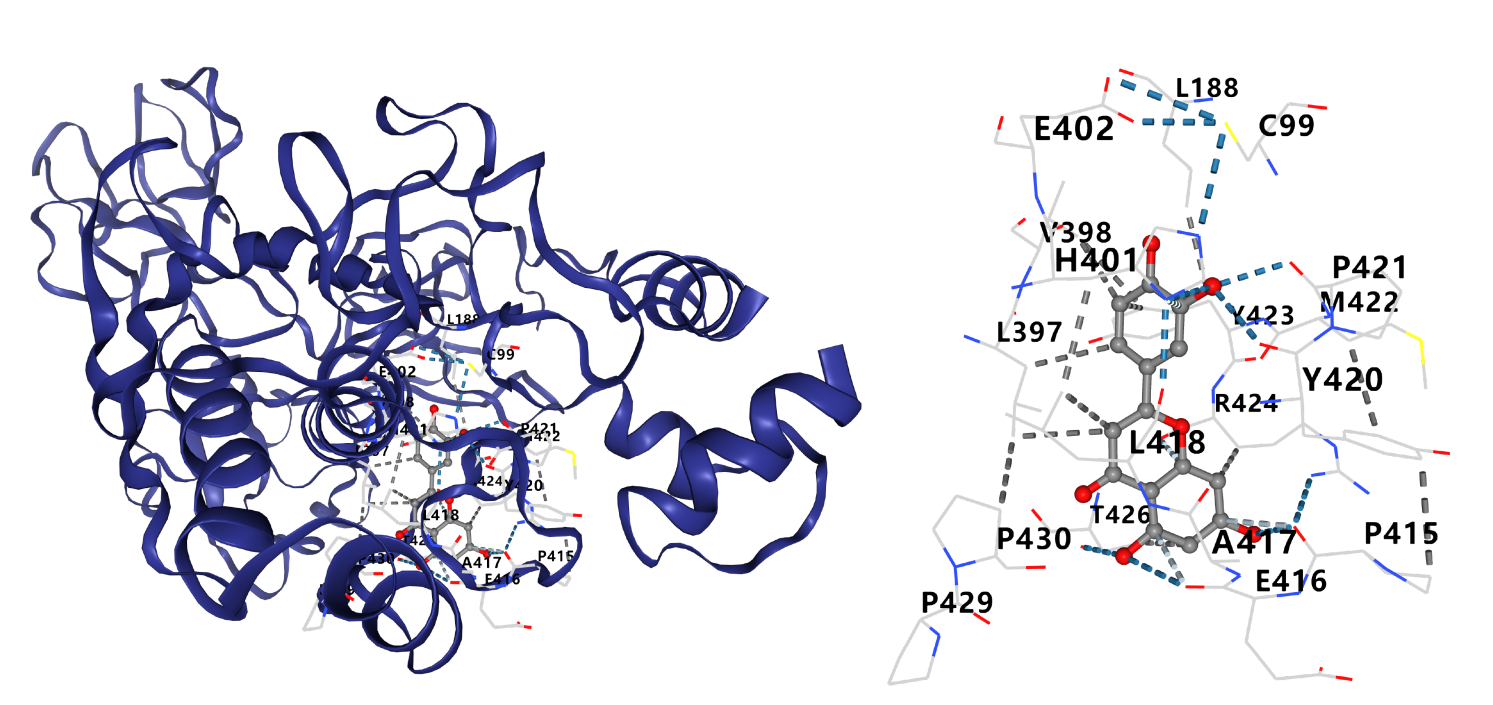


**Supplementary Figure 2.** The figure shows the visual display of the docking result between luteolin and MMP9 molecules, indicating that the docking binding energy is -10.1 kcal/mol. It should be noted that this analysis is predictive and has not been functionally validated; therefore, these findings are hypothesis-generating and intended only for guidance in future mechanistic research.

## Supplementary Tables

**Supplementary Table 1.** Summary of the GEO datasets used for bioinformatics analysis, including dataset accession numbers, sample labels, disease status, species, tissue source, and technology platform.

| **Dataset** | **Sample Number** | **Sample Lable** | **Disease stage** | **Species** | **Tissue Source** | **Technology Platform** |
| --- | --- | --- | --- | --- | --- | --- |
| GSE114007 | GSM3130531 | Normal_Cart_2_2 | Normal | Homo sapiens | knee cartilage | Illumina HiSeq 2000 |
|  | GSM3130532 | Normal_Cart_3_3 | Normal | Homo sapiens | knee cartilage | Illumina HiSeq 2000 |
|  | GSM3130533 | Normal_Cart_4_4 | Normal | Homo sapiens | knee cartilage | Illumina HiSeq 2000 |
|  | GSM3130534 | Normal_Cart_5_5 | Normal | Homo sapiens | knee cartilage | Illumina HiSeq 2000 |
|  | GSM3130535 | Normal_Cart_6_6 | Normal | Homo sapiens | knee cartilage | Illumina HiSeq 2000 |
|  | GSM3130536 | Normal_Cart_7_3 | Normal | Homo sapiens | knee cartilage | Illumina HiSeq 2000 |
|  | GSM3130537 | Normal_Cart_9_7 | Normal | Homo sapiens | knee cartilage | Illumina HiSeq 2000 |
|  | GSM3130538 | Normal_Cart_10_8 | Normal | Homo sapiens | knee cartilage | Illumina HiSeq 2000 |
|  | GSM3130539 | OA_Cart_1_7 | OA | Homo sapiens | knee cartilage | Illumina HiSeq 2000 |
|  | GSM3130540 | OA_Cart_2_8 | OA | Homo sapiens | knee cartilage | Illumina HiSeq 2000 |
|  | GSM3130541 | OA_Cart_3_9 | OA | Homo sapiens | knee cartilage | Illumina HiSeq 2000 |
|  | GSM3130542 | OA_Cart_4_10 | OA | Homo sapiens | knee cartilage | Illumina HiSeq 2000 |
|  | GSM3130543 | OA_Cart_5_5 | OA | Homo sapiens | knee cartilage | Illumina HiSeq 2000 |
|  | GSM3130544 | OA_Cart_6_1 | OA | Homo sapiens | knee cartilage | Illumina HiSeq 2000 |
|  | GSM3130545 | OA_Cart_7_2 | OA | Homo sapiens | knee cartilage | Illumina HiSeq 2000 |
|  | GSM3130546 | OA_Cart_8_5 | OA | Homo sapiens | knee cartilage | Illumina HiSeq 2000 |
|  | GSM3130547 | OA_Cart_9_6 | OA | Homo sapiens | knee cartilage | Illumina HiSeq 2000 |
|  | GSM3130548 | OA_Cart_10_9 | OA | Homo sapiens | knee cartilage | Illumina HiSeq 2000 |
|  | GSM3130549 | normal_01 | Normal | Homo sapiens | knee cartilage | Illumina NextSeq 500 |
|  | GSM3130550 | normal_02 | Normal | Homo sapiens | knee cartilage | Illumina NextSeq 500 |
|  | GSM3130551 | normal_03 | Normal | Homo sapiens | knee cartilage | Illumina NextSeq 500 |
|  | GSM3130552 | normal_04 | Normal | Homo sapiens | knee cartilage | Illumina NextSeq 500 |
|  | GSM3130553 | normal_05 | Normal | Homo sapiens | knee cartilage | Illumina NextSeq 500 |
|  | GSM3130554 | normal_06 | Normal | Homo sapiens | knee cartilage | Illumina NextSeq 500 |
|  | GSM3130555 | normal_07 | Normal | Homo sapiens | knee cartilage | Illumina NextSeq 500 |
|  | GSM3130556 | normal_08 | Normal | Homo sapiens | knee cartilage | Illumina NextSeq 500 |
|  | GSM3130557 | normal_09 | Normal | Homo sapiens | knee cartilage | Illumina NextSeq 500 |
|  | GSM3130558 | normal_10 | Normal | Homo sapiens | knee cartilage | Illumina NextSeq 500 |
|  | GSM3130559 | OA_01 | OA | Homo sapiens | knee cartilage | Illumina NextSeq 500 |
|  | GSM3130560 | OA_02 | OA | Homo sapiens | knee cartilage | Illumina NextSeq 500 |
|  | GSM3130561 | OA_03 | OA | Homo sapiens | knee cartilage | Illumina NextSeq 500 |
|  | GSM3130562 | OA_04 | OA | Homo sapiens | knee cartilage | Illumina NextSeq 500 |
|  | GSM3130563 | OA_05 | OA | Homo sapiens | knee cartilage | Illumina NextSeq 500 |
|  | GSM3130564 | OA_06 | OA | Homo sapiens | knee cartilage | Illumina NextSeq 500 |
|  | GSM3130565 | OA_07 | OA | Homo sapiens | knee cartilage | Illumina NextSeq 500 |
|  | GSM3130566 | OA_08 | OA | Homo sapiens | knee cartilage | Illumina NextSeq 500 |
|  | GSM3130567 | OA_09 | OA | Homo sapiens | knee cartilage | Illumina NextSeq 500 |
|  | GSM3130568 | OA_10 | OA | Homo sapiens | knee cartilage | Illumina NextSeq 500 |
| GSE169077 | GSM5176138 | N2 | Normal | Homo sapiens | knee cartilage | Affymetrix Human Genome U133A Array |
|  | GSM5176139 | N4 | Normal | Homo sapiens | knee cartilage | Affymetrix Human Genome U133A Array |
|  | GSM5176140 | N6 | Normal | Homo sapiens | knee cartilage | Affymetrix Human Genome U133A Array |
|  | GSM5176141 | N7 | Normal | Homo sapiens | knee cartilage | Affymetrix Human Genome U133A Array |
|  | GSM5176142 | N8 | Normal | Homo sapiens | knee cartilage | Affymetrix Human Genome U133A Array |
|  | GSM5176143 | OA2 | OA | Homo sapiens | knee cartilage | Affymetrix Human Genome U133A Array |
|  | GSM5176144 | OA3 | OA | Homo sapiens | knee cartilage | Affymetrix Human Genome U133A Array |
|  | GSM5176145 | OA8 | OA | Homo sapiens | knee cartilage | Affymetrix Human Genome U133A Array |
|  | GSM5176146 | OA9 | OA | Homo sapiens | knee cartilage | Affymetrix Human Genome U133A Array |
|  | GSM5176147 | OA10 | OA | Homo sapiens | knee cartilage | Affymetrix Human Genome U133A Array |
|  | GSM5176148 | OA11 | OA | Homo sapiens | knee cartilage | Affymetrix Human Genome U133A Array |
| GSE255460 | GSM8072834 | C1_Condition_Control_Rep1 | Normal | Homo sapiens | knee cartilage | Illumina NovaSeq 6000 |
|  | GSM8072835 | C2_Condition_Control_Rep2 | Normal | Homo sapiens | knee cartilage | Illumina NovaSeq 6000 |
|  | GSM8072836 | C3_Condition_Control_Rep3 | Normal | Homo sapiens | knee cartilage | Illumina NovaSeq 6000 |
|  | GSM8072837 | OA1-1_Condition_NWB_Rep1 | OA | Homo sapiens | knee cartilage | Illumina NovaSeq 6000 |
|  | GSM8072838 | OA1-2_Condition_WB_Rep1 | OA | Homo sapiens | knee cartilage | Illumina NovaSeq 6000 |
|  | GSM8072839 | OA2-1_Condition_NWB_Rep2 | OA | Homo sapiens | knee cartilage | Illumina NovaSeq 6000 |
|  | GSM8072840 | OA2-2_Condition_WB_Rep2 | OA | Homo sapiens | knee cartilage | Illumina NovaSeq 6000 |
|  | GSM8072841 | OA3-1_Condition_NWB_Rep3 | OA | Homo sapiens | knee cartilage | Illumina NovaSeq 6000 |
|  | GSM8072842 | OA3-2_Condition_WB_Rep3 | OA | Homo sapiens | knee cartilage | Illumina NovaSeq 6000 |
|  | GSM8072843 | OA4-1_Condition_NWB_Rep4 | OA | Homo sapiens | knee cartilage | Illumina NovaSeq 6000 |
|  | GSM8072844 | OA4-2_Condition_WB_Rep4 | OA | Homo sapiens | knee cartilage | Illumina NovaSeq 6000 |
|  | GSM8072845 | OA5-1_Condition_NWB_Rep5 | OA | Homo sapiens | knee cartilage | Illumina NovaSeq 6000 |
|  | GSM8072846 | OA5-2_Condition_WB_Rep5 | OA | Homo sapiens | knee cartilage | Illumina NovaSeq 6000 |
|  | GSM8072847 | OA6-1_Condition_NWB_Rep6 | OA | Homo sapiens | knee cartilage | Illumina NovaSeq 6000 |
|  | GSM8072848 | OA6-2_Condition_WB_Rep6 | OA | Homo sapiens | knee cartilage | Illumina NovaSeq 6000 |
|  | GSM8072849 | OA7-1_Condition_NWB_Rep7 | OA | Homo sapiens | knee cartilage | Illumina NovaSeq 6000 |
|  | GSM8072850 | OA7-2_Condition_WB_Rep7 | OA | Homo sapiens | knee cartilage | Illumina NovaSeq 6000 |
|  | GSM8072851 | OA8-1_Condition_NWB_Rep8 | OA | Homo sapiens | knee cartilage | Illumina NovaSeq 6000 |
|  | GSM8072852 | OA8-2_Condition_WB_Rep8 | OA | Homo sapiens | knee cartilage | Illumina NovaSeq 6000 |

**Supplementary Table S1**

| MMP9-F | GATCCCCAGAGCGTTACTCG | |
| --- | --- | --- |
| MMP9-R | GTTGTGGAAACTCACACGCC | |
| MMP2-F | GGTGGCAATGGAGATGGACA |  |
| MMP2-R | CCCGGTCATAATCCTCGGTG |  |
| SPP1-F | CCAGCCAAGGACCAACTACA |  |
| SPP1-R | AGTGTTTGCTGTAATGCGCC |  |
| GAPDH-F | GGCCGGAGACGAATGGAAATTA |  |
| GAPDH-R | CCAAATCCGTTCACACCGAC |  |

**Supplementary Table S2**

| **Component** | **Volume** |
| --- | --- |
| 5x Reaction Buffer | 4 ul |
| Primer | 1 ul |
| SweScript RT I Enzyme Mix | 1 ul |
| Total RNA | 2 ug |
| Nuclease-Free Water | Add to 20 ul |

**Supplementary Table S3**

| **Temperature** | **Time** |
| --- | --- |
| 25℃ | 5 min |
| 50℃ | 15 min |
| 85℃ | 5 s |
| 4℃ | hold |

**Supplementary Table S4**

| **Component** | **Volume** |
| --- | --- |
| cDNA | 3 ul |
| 2x Universal Blue SYBR Green qPCR Master Mix | 5 ul |
| Forward primer (10 µM) | 1 ul |
| Reverse primer (10 µM) | 1 ul |

**Supplementary Table S5**

|  | **Temperature** | **Time** |
| --- | --- | --- |
| Initial denaturation | 95℃ | 1 min |
| Denaturation | 95℃ | 20 s |
| Annealing | 55℃ | 20 s |
| Extension | 72℃ | 30 s |
